# Identification of a novel prognostic and therapeutic prediction model in clear cell renal carcinoma based on Renin-angiotensin system related genes

**DOI:** 10.3389/fendo.2025.1521940

**Published:** 2025-03-03

**Authors:** Qinzheng Chang, Shuo Zhao, Jiajia Sun, Wei Guo, Lin Yang, Laiyuan Qiu, Nianzhao Zhang, Yidong Fan, Jikai Liu

**Affiliations:** Department of Urology, Qilu Hospital of Shandong University, Jinan, Shandong, China

**Keywords:** risk model, ccRCC, renin angiotensin system, SLC6A19, CPT1a

## Abstract

**Background:**

Clear cell renal cell carcinoma is the most predominant type of renal malignancies, characterized by high aggressiveness and probability of distant metastasis. Renin angiotensin system (RAS) plays a crucial role in maintaining fluid balance within the human body, and its involvement in tumorigenesis is increasingly being uncovered, while its role in ccRCC remains unclear.

**Methods:**

WGCNA was used to identify RAS related genes. Machine learning was applied to screen hub genes for constructing risk model, E-MTAB-1980 dataset was used for external validation. Transwell and CCK8 assays were used to investigate the impact of SLC6A19 to ccRCC cells.

**Results:**

SLC6A19, SLC16A12 and SMIM24 were eventually screened to construct risk model and the predictive efficiency for prognosis was validated by internal and external cohorts. Moreover, the differences were found in pathway enrichment, immune cell infiltration, mutational landscapes and drug prediction between high and low risk groups. Experimental results indicated that SLC6A19 could inhibit invasion and proliferation of ccRCC cells and GSEA pinpointed that SLC6A19 was intimately correlated with fatty acid metabolism and CPT1A.

**Conclusion:**

The risk model based on the three RAS-related genes have a robust ability to predict the prognosis and drug sensitivity of ccRCC patients, further providing a valid instruction for clinical care.

## Introduction

Renal cell carcinoma (RCC) is one of the most common malignancies in the urogenital system and ranks among the 10 most prevalent cancers worldwide (1). The main histologic subtypes of RCC include clear cell renal cell carcinoma (ccRCC), papillary renal cell carcinoma (pRCC), and chromophobe renal cell carcinoma (chRCC), with ccRCC accounting for approximately 70% of all RCC cases diagnosed (2). Patients with early-stage ccRCC typically achieve complete or partial remission through nephrectomy; however, up to a third of these cases may still progress to advanced stages and develop distant metastasis (3, 4). ccRCC is responsible for the majority of kidney cancer deaths, and the 5-year overall survival rate is less than 20% (5). Moreover, due to the limited sensitivity of ccRCC to radiotherapy and chemotherapy, finding effective treatments for patients with distant metastasis poses a significant challenge. Although targeted therapies, including VEGFR inhibitors, mTOR inhibitors (such as everolimus), and immune checkpoint inhibitors, have significantly improved the prognosis for advanced patients, some patients are prone to developing resistance to these drugs and thus do not benefit from them (4, 6). In line with advancements in genomic sequencing technology, predictive models for outcomes and drug sensitivity in ccRCC should be developed at the genetic level.

The Renin-Angiotensin System (RAS) is a crucial biochemical pathway that maintains electrolyte balance, arterial blood pressure, and extracellular volume (7). The RAS primarily consists of angiotensinogen (AGT), renin, angiotensin-converting enzyme 1 (ACE1), and angiotensin-converting enzyme 2 (ACE2). The initial step in the RAS cascade involved renin secreted by the kidney, which cleaves angiotensinogen, primarily synthesized by hepatocytes, to form the decapeptide angiotensin I (Ang I). Ang I is then further cleaved by ACE1 to produce the octapeptide Ang II (8). Ang II, the final effector of the RAS, modulates blood pressure by specifically binding to angiotensin II receptor type 1 (AGTR1) and angiotensin II receptor type 2 (AGTR2) (9). A vital alternative pathway within the RAS is the conversion of Ang II to Ang(1–7) by ACE2, which counteracts the effects of Ang II by acting on the Mas receptor(MASR) (10). Currently, there is a growing body of research exploring the interaction between the RAS and various neoplasms, such as pancreatic cancer, glioblastoma, endometrial cancer, lung cancer, and breast cancer (7, 8, 11, 12). The core components of the RAS may influence tumor angiogenesis, metastasis, and apoptosis by regulating multiple signaling pathways, and RAS inhibitors are anticipated as potential novel agents for the treatment of advanced malignancies (13, 14). Several studies have also highlighted the crosstalk between the RAS and renal disorders, including renal cancer, given that the kidney is one of the primary target organs of the RAS (15, 16). Therefore, in this study, we aim to develop a robust predictive model for ccRCC using diverse bioinformatics algorithms based on the RAS pathway.

SLC6A19 is a Na+-coupled transporter for neutral amino acids, plays a pivotal role in the intestinal absorption of amino acids derived from dietary proteins and in the renal reabsorption of circulating amino acids filtered at the glomerulus (17). It is instrumental in sustaining amino acid homeostasis in humans. Loss-of-function mutations in this transporter can precipitate a range of amino acid metabolism disorders, with the most prevalent being Hartnup disease (18). In this study, we revealed that SLC6A19 acts as a significant gene associated with the RAS pathway, playing a crucial role in regulating the progression of ccRCC.

## Materials and methods

### Data collection

The expression profiles of bulk RNA-seq for ccRCC were downloaded from TCGA database (https://portal.gdc.cancer.gov/, 532 primary tumor and 72 normal samples), GSE53757(72 tumor and 72 normal samples) dataset in GEO database(https://www.ncbi.nlm.nih.gov/geo/) and E-MTAB-1980 cohort(101 tumor samples) in ArrayExpress database(https://www.ebi.ac.uk/arrayexpress/).For bulk RNA-seq in TCGA, the log2(TPM+1) normalization of raw count matrix was adopted. The clinical and survival data of ccRCC was collected from TCGA and ArrayExpress databases. The scRNA-seq data was obtained from GSE159115 dataset containing 7 ccRCC and 6 normal samples in GEO database.

### Calculation of RAS score

A gene set of the Renin-Angiotensin System (RAS), comprising 17 signature genes, was extracted from the “c2.cp.KEGG_RENIN_ANGIOTENSIN_SYSTEM” collection in MsigDB database(https://www.gsea-msigdb.org/gsea/msigdb/index.jsp) and single sample gene set enrichment analysis(ssGAEA) was utilized to calculate RAS score based on expression level of 17 RAS signature genes in TCGA database via “GSVA” package.

### Weighted gene co-expression network analysis

Weighted Gene Co-expression Network Analysis (WGCNA) was utilized to identify co-expression gene modules and to screen for the module most correlated with the RAS score. The analysis was based on an expression matrix downloaded from the TCGA-KIRC item. Initially, we calculated the median absolute deviation (MAD) for each gene and selected the top 5000 for further analysis. This step was performed to filter out hypovariant genes. Subsequently, a scale-free co-expression network was constructed using the ‘WGCNA’ package with a soft threshold parameter of 6, resulting in the confirmation of seven modules. Finally, a correlation analysis was conducted between the gene modules and the RAS score. Genes within the module that showed the highest relevance to the RAS score were selected for subsequent analysis.

### Differential expression analysis

Differentially expressed genes (DEG) were identified through “limma” package and the selection criteria for differential genes were an absolute value of the log2 fold change (|log2 FC|) greater than 1 and an adjusted p-value less than 0.05.

### Screening flow of key genes for prognosis analysis

We performed an intersection of genes within the green module identified by WGCNA analysis with those that were differentially expressed between tumor and normal groups, as well as between groups with high and low RAS scores, using the Venn diagram approach. The genes resulting from this intersection were then subjected to univariate Cox regression analysis to preliminarily identify potential prognostic markers. Subsequently, two machine learning algorithms, the Least Absolute Shrinkage and Selection Operator (Lasso) regression and the Random Forest(RF) analysis, were employed to further identify more robust prognostic genes depending on “glmnet” and “randomForestSRC “ R packages respectively. For Lasso regression, the model was specified with alpha = 1, the family parameter was set to “cox” and the nlambda parameter was set to 100.To determine the optimal regularization parameter (lambda), we conducted cross-validation using the “cv.glmnet” function and we considered the lambda value corresponding to the 1-standard error rule (lambda.1se).With regard to RF, we use the “rfsrc” function to fit a random forest model to survival data and the top 20 most important genes are selected for further analysis. Eventually, by intersecting the gene sets obtained from two above algorithms, we successfully pinpointed the key genes.

### Construction and validation of risk model

We randomly divide the 532 patients in TCGA-KIRC into training and testing cohorts in a 1:1 ratio. The key genes selected in previous step were firstly included in multivariate cox regression analysis based on expression and survival data in training cohort for constructing predictive risk model utilizing “survminer” package and the genes with following coefficient were determined while the genes lacking coefficient were excluded. The risk score of each patient was eventually calculated following the formula: Risk score= ∑^n^_i_ Coefi * Expression (X_i_). The “Coefi” was defined as the coefficient of each gene and “Expression (X_i_)” was defined as the expression value of each gene. To evaluate the predictive efficiency of risk model, K-M analysis, time-dependent ROC analysis and principal component analysis(PCA) were performed. Furthermore, we calculated the risk scores for patients in both the testing and entire TCGA cohorts to internally validate the predictive accuracy of our risk model. For external validation, we utilized the E-MTAB-1980 cohort.

### Functional enrichment analysis

Kyoto Encyclopedia of Genes and Genomes (KEGG) and Gene Ontology (GO) pathway enrichment analyses were conducted for differentially expressed genes (DEGs) identified between the high and low-risk groups, utilizing the ‘clusterProfiler’ package. Additionally, Gene Set Enrichment Analysis (GSEA) was performed on DEGs between different risk groups and DEGs between groups with high and low SLC6A19 expression. For the GSEA, ‘Hallmark’ gene sets were downloaded from the MsigDB database and analyzed using the ‘clusterProfiler’ package.

### Immune infiltration analysis

The CIBERSORT algorithm was used to calculate the immune infiltration fractions of 22 immune cells in both high and low-risk groups. The signatures for these 28 immune cells and 13 immune-related pathways, along with their associated genes, were derived from the articles with PMID: 28052254 and PMID: 30594216, respectively (19, 20), ssGSEA analysis was conducted to calculate their immune scores.

### Somatic mutation analysis and drug sensitivity analysis

Somatic mutation data of ccRCC was downloaded from the TCGA database using the ‘TCGAbiolinks’ package, with the data category selected as ‘Simple Nucleotide Variation’. To visualize the mutation status of high-frequency mutant genes, waterfall charts were generated using the ‘maftools’ package.

Drug sensitivity analysis was performed using the “oncoPredict” package. The operating principle involves establishing a training model based on existing cell line expression matrices and drug sensitivity data, which can be obtained from databases such as GDSC, CTRP, or CCLE. This model is then used to make predictions on new expression matrices. Drug sensitivity was quantified as the half-maximal inhibitory concentration (IC50), with smaller values indicating greater sensitivity to the drug.

### Single-cell RNA-seq analysis

The single-cell RNA sequencing (scRNA-seq) data were imported into Seurat objects using the Seurat R package, facilitating advanced data analysis and visualization. We performed cell quality control analysis and cells that failed to meet the criteria (nFeature_RNA > 200 & nFeature_RNA < 2500 & mitochondrial genes<5%) were excluded (**Supplementary Figure 4**). We employed the “LogNormalize” method to standardize the seurat object and the top 2000 highly variable genes were identified using normalized data. Principal component analysis (PCA) was conducted using the RunPCA function, and batch effects were removed using the harmony method. The top 10 principal components were selected based on the ElbowPlot function for cell clustering utilizing the FindNeighbors, FindClusters, and RunUMAP functions. We utilize the FindAllMarkers function to systematically identify differentially expressed genes that serve as markers for each distinct cell cluster. The cell types were preliminarily annotated using the SingleR R package and further verified by manual tagging using classic cell markers through Cell Marker2.0 website.

### Cell lines and culture

The 786O and A498 cancer cell lines were purchased from CellSource China. Their mycoplasma contamination status was routinely checked before experiments. All cell lines were cultured in media containing 10% fetal bovine serum (ExCell Bio, China) and 1% penicillin and streptomycin (KeyGen Biotech, China). They were incubated at 37°C with 5% CO2.

### Cell transfection

To perform cell transfection, seed cells until the fusion degree reaches 30%–50%. Prepare a transfection mixture by mixing 4 µg of plasmid DNA or 20 nM siRNA with 200 µL of transfection buffer and add 8 µL of jetPRIME reagent (Polyplus transfection, 101000046). After thorough mixing, let it stand for 10 minutes to form complexes. Add the mixture to the culture dish, shake gently, and incubate at 37°C with 5% CO_2_. Monitor cell growth and morphology. After 48 or 72 hours, harvest cells to measure RNA and protein expression levels.

### Cell counting kit-8 assay

The cells were distributed into 96-well plates at an appropriate density, typically ranging from 1x10^4 to 1x10^5 cells per well, and cultured under conditions of 37°C and 5% CO2. At 24, 48 and 72 hours post-cell culture initiation, 10 µL of CCK-8 reagent was added to each well. The plates were then allowed to react for 2 hours before measuring the absorbance values at 450 nm at each of these time points to evaluate the cell viability.

### Transwell assay

The transwell assay was used to estimate the cell migration and invasion abilities of various groups. A suspension containing 5x10^5 cells resuspended in serum-free medium was inoculated into the upper chamber. For the migration assay, the chamber was without matrix gel; for the invasion assay, it was filled with matrix gel. Then, 500 µL of complete medium was added to the lower chamber. The cells were cultured for 24 hours at 37°C, 5% CO2, and 90% humidity. After incubation, the cells that had migrated to the lower surface of the membrane were fixed for 30 minutes using 4% paraformaldehyde and subsequently stained with crystal violet for 10 minutes. Finally, the wells were washed three times to remove non-migrated cells, and the migrated cells were observed and quantified using a microscope and ImageJ software (National Institutes of Health, Bethesda, MD, USA).

### Statistical analyses

All statistical analyses were conducted using R software (version 4.3.0) and GraphPad Prism (Version 9.0). For comparing continuous variables that follow a normal distribution, Student’s t-test was employed, while the Wilcoxon test was used for non-parametric variables. The Spearman correlation analysis was applied to determine the correlation coefficient between two continuous variables. Finally, a p-value of less than 0.05 was considered statistically significant.

## Results

### Expression and clinical role of RAS signature genes in ccRCC

**Figure 1:**
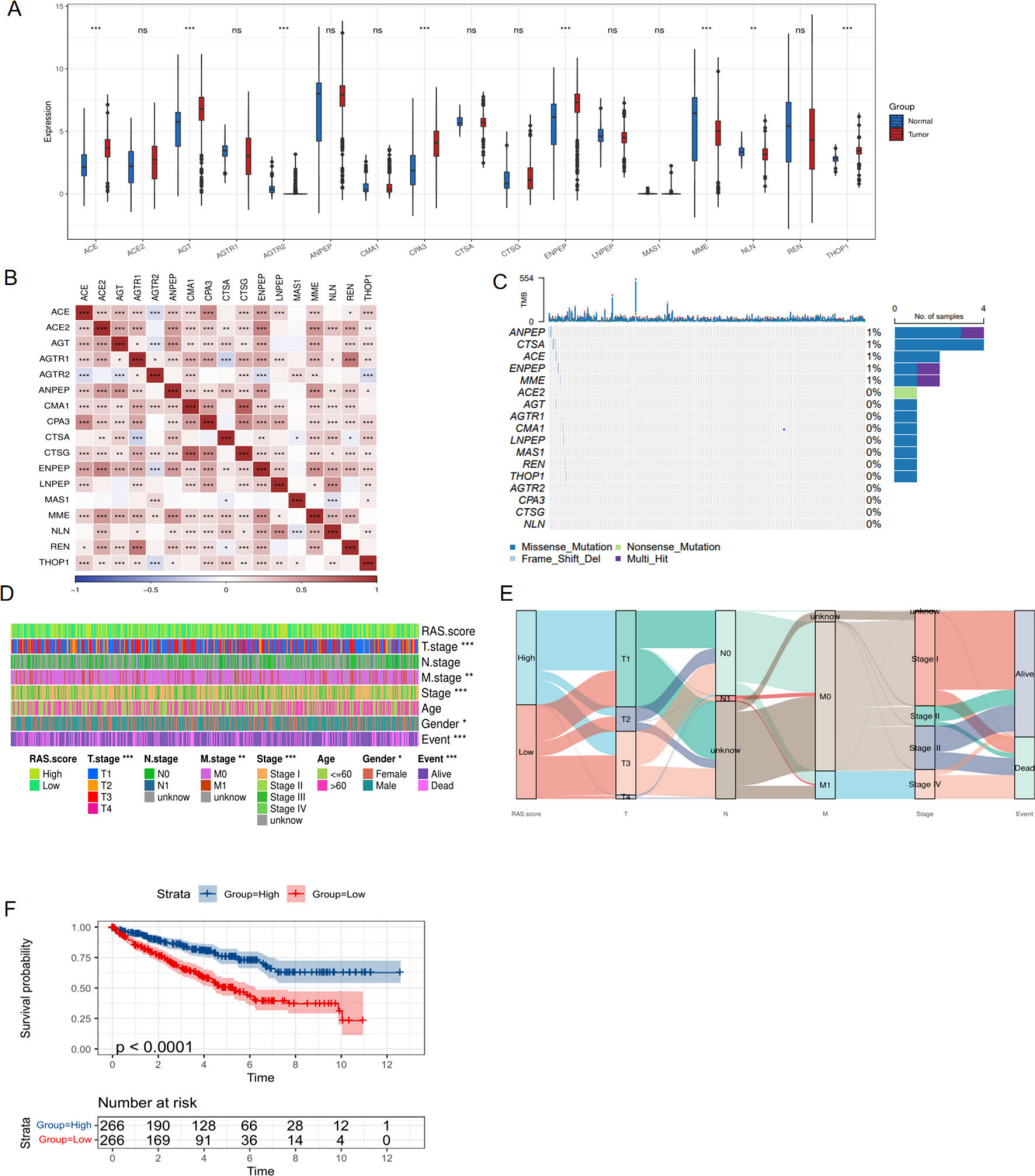
Expression and clinical role of RAS signature genes based on TCGA database. **(A)** Differential expression of RAS signature genes between tumor and normal tissue of KIRC cohort; **(B)** Correlation heatmap showing the relationship between RAS signature genes; **(C)** The mutation of RAS signature genes in KIRC cohort; **(D)** Heatmap showing the correlation between RAS score and clinical parameters; **(E)** The Sankey diagram shows the connection degree between the RAS score and clinical parameters of KIRC; **(F)** Kaplan-Meier curve showing the impact of RAS score on survival of ccRCC patients. *p < 0.05, **p < 0.01, ***p < 0.005. ns, not significant.

The expression profile of 17 signature genes was extracted from the TCGA database. Initially, we compared the expression levels of these 17 genes in tumor and normal tissues of ccRCC patients and observed that the majority of these genes exhibited increased expression in tumor tissue (**Figure 1A**). However, from the Kaplan-Meier curves of these 17 genes, it was observed that most genes were favorable to the overall survival of ccRCC patients (**Supplementary Figure 1**). To further investigate the relationships among these RAS signature genes, we conducted a correlation analysis and generated a heatmap, which overall revealed a positive correlation trend (**Figure 1B**). Additionally, the waterfall plot revealed the mutation status of the 17 RAS signature genes in ccRCC, with ANPEP, CTSA, ACE, ENPEP, and MME being the only genes with a significantly higher mutation rate (**Figure 1C**). Subsequently, the RAS score was determined using the ssGSEA algorithm to evaluate the collective impact of these 17 genes on ccRCC. The heatmap analysis indicated that the RAS score was intimately correlated with a range of clinical parameters, including T stage, M stage, AJCC stage, gender, and survival events (**Figure 1D**). Sankey plot was also drawn and demonstrated that a high RAS score mainly indicates earlier stage and alive events for ccRCC patients (**Figure 1E**). To verify the prognostic role of the RAS score, we performed Kaplan-Meier analysis, and the results showed that a higher score significantly predicted a better prognosis for ccRCC patients (**Figure 1F**, P<0.001).

### Identification of the RAS related genes

**Figure 2:**
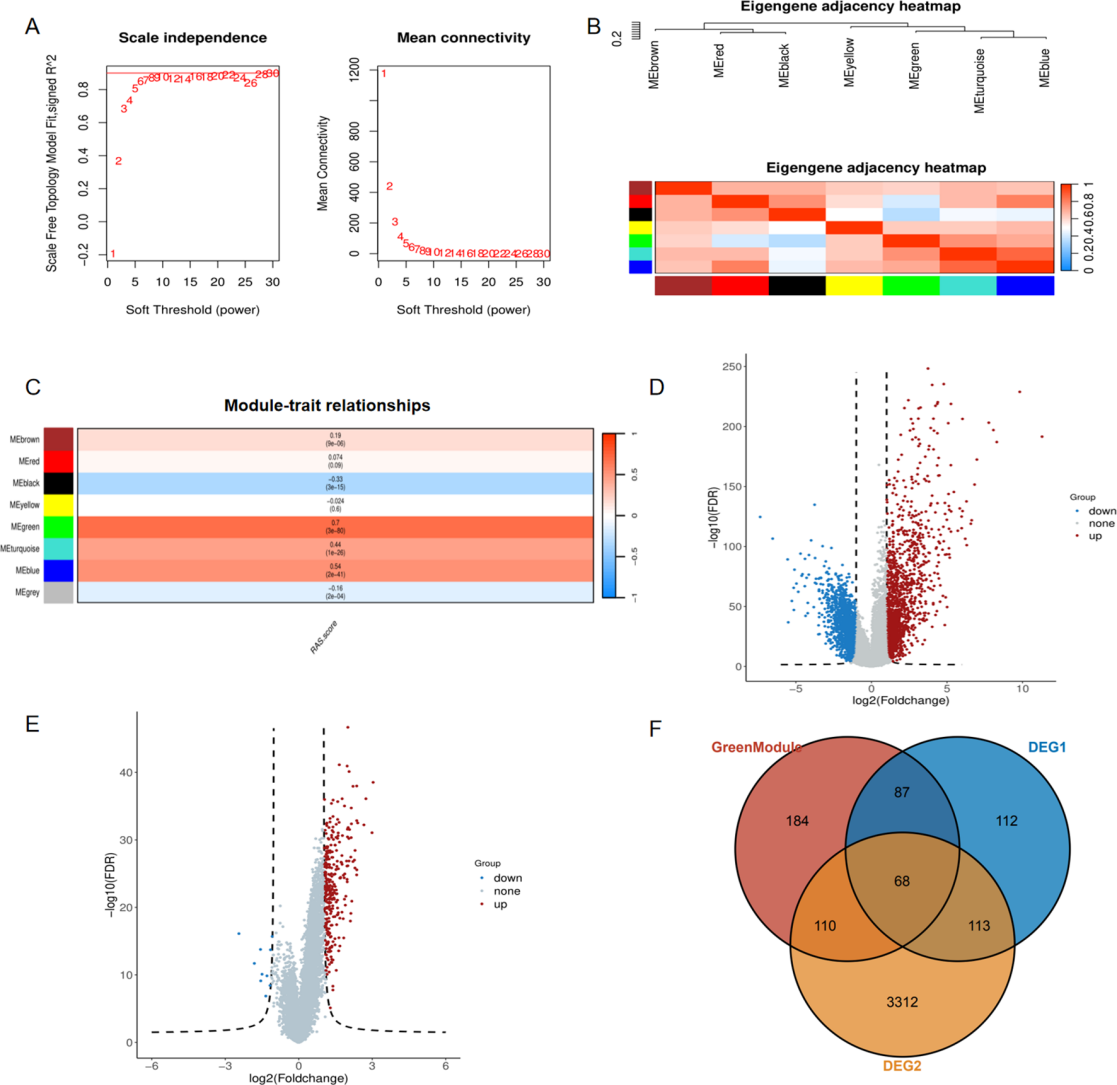
Identification of the RAS related genes via weighted gene coexpression network analysis. **(A)** The scale-free fit index(left panel) and the average connectivity of soft threshold power(right panel) are confirmed for KIRC cohort; **(B)** Eigengene adjacency heatmap showing correlations between seven modules; **(C)** The correlation between modules and RAS score; **(D)** Differentially expressed genes between tumor and normal tissue of ccRCC; **(E)** Differentially expressed genes between high RAS score and low RAS score groups. **(F)** Venn diagram showing common genes between green module and two kinds of DEGs.

To explore the underlying function of the RAS pathway in ccRCC, we performed WGCNA to identify the gene module that is highly related to the RAS score. Initially, we selected the top 5,000 genes with the highest absolute median difference ranking from the 532 tumor samples of the TCGA-KIRC cohort. The soft threshold power was set to 6 to achieve a scale-free topology (scale-free R2 = 0.9) (**Figure 2A**). Subsequently, co-expressed genes were clustered into the same module, and seven modules were eventually confirmed. The relationship among these modules was presented in an eigengene adjacency heatmap (**Figure 2B**). We interacted seven eigengene modules with RAS score and screened the most correlated module with phenotype, result showed that the MEgreen module exhibited statistically significant positive correlation with RAS score (**Figure 2C**, R = 0.7, P = 3e-80).We then conducted differential gene analysis and filtered two types of differentially expressed genes, one between the tumor and normal groups (**Figure 2D**), and the other between the high and low RAS score groups (**Figure 2E**). In the end, we intersect two kinds of DEGs with genes in MEgreen module and 68 RAS related genes(RRGs) were identified (**Figure 2F**).

### Construction and validation of prognostic risk model based on RRG

**Figure 3:**
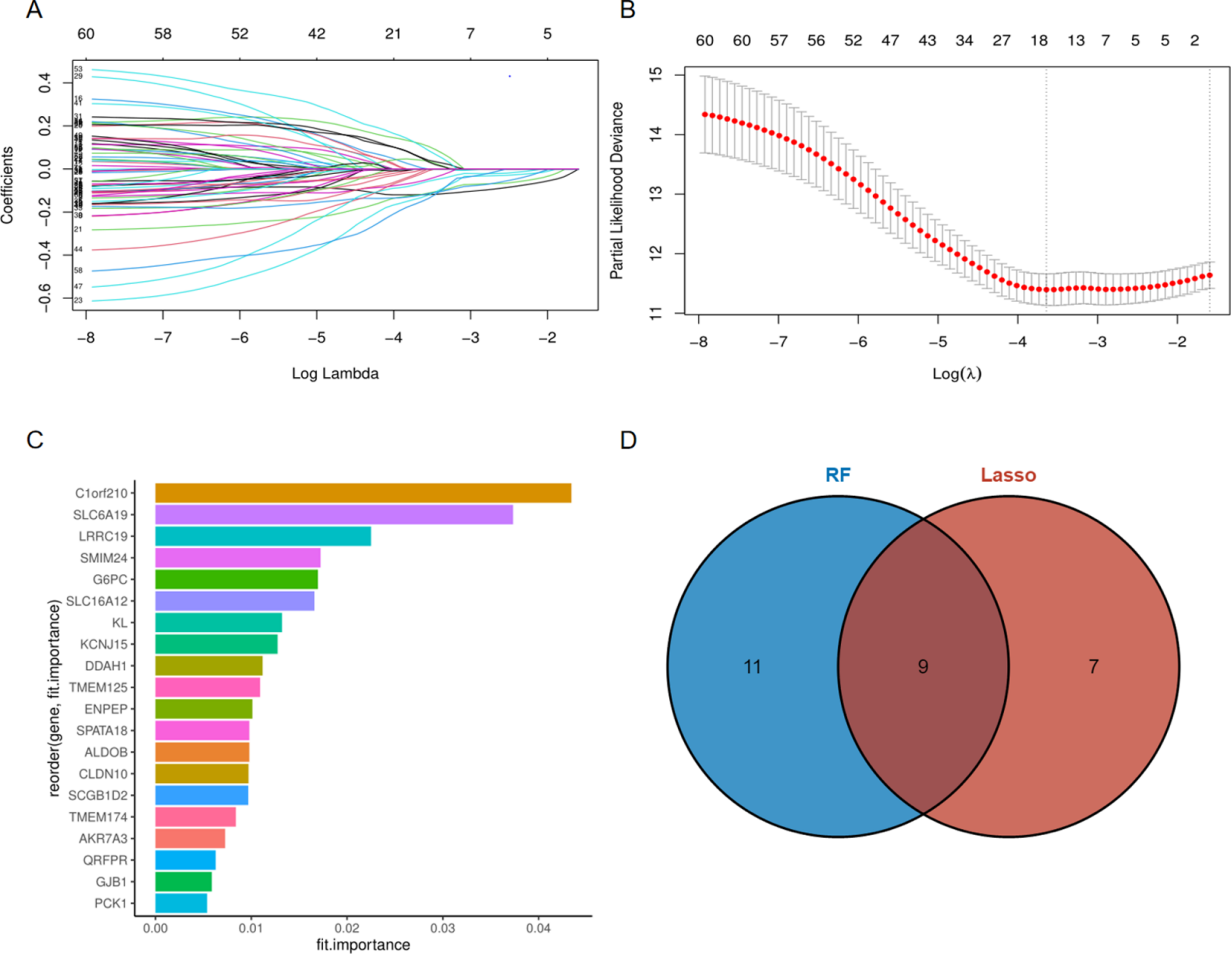
Selection of key prognostic genes. **(A)** Identification of prognostic genes through Lasso regression; **(B)** Cross validation of selected genes; **(C)** Identification of prognostic genes through RandomForest; **(D)** Common genes screened by Lasso regression and RandomForest.

**Figure 4:**
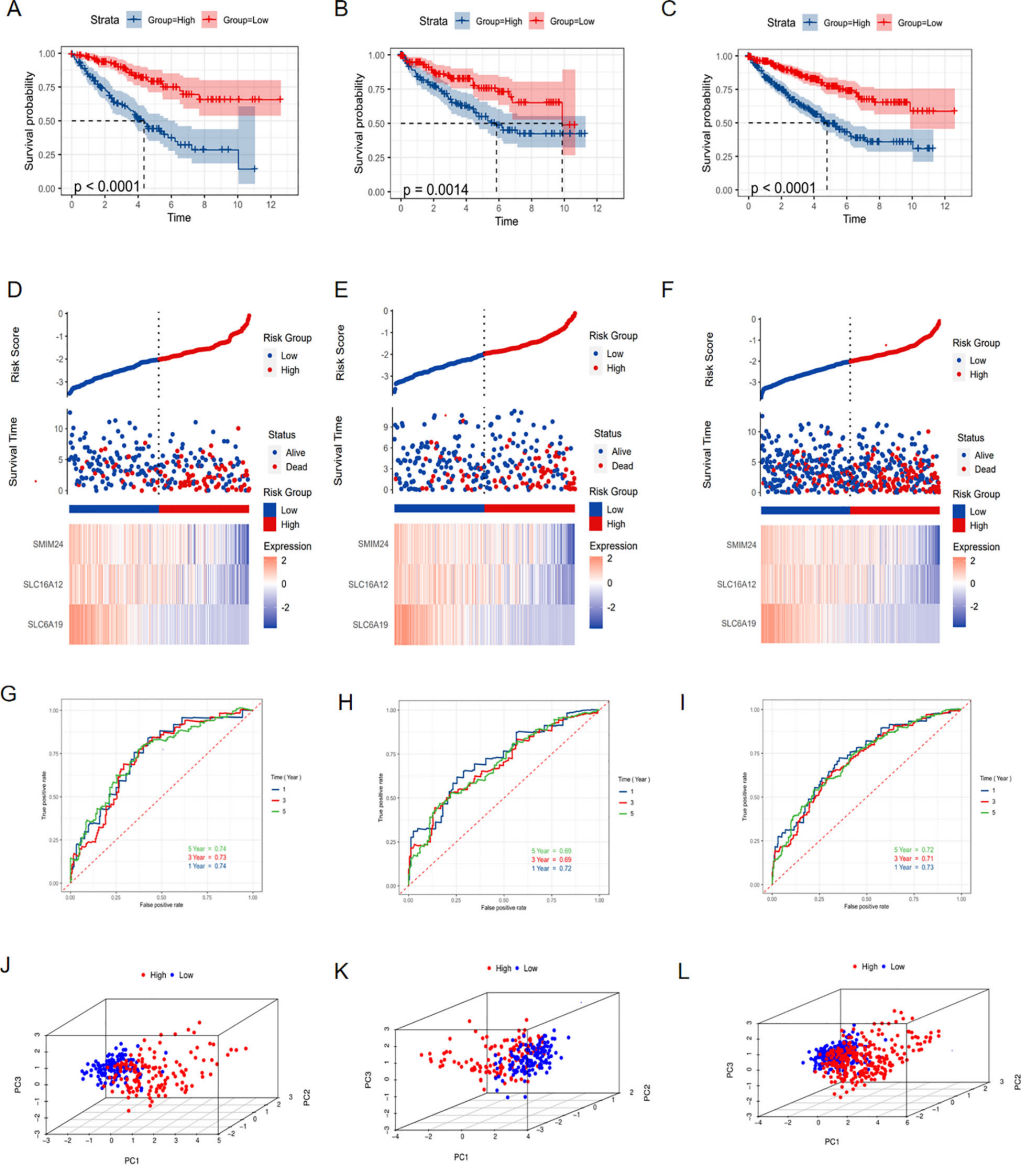
Construction and validation of prognostic model. Kaplan-Meier curve showing the different overall survival between high and low risk score groups in **(A)** training set, **(B)** testing set and **(C)** whole TCGA-KIRC cohort. Risk distribution map showing the correlation between risk score and outcome events in **(D)** training set, **(E)** testing set and **(F)** whole TCGA-KIRC cohort. ROC curve showing the predictive value of risk model for 1-, 3-, and 5- year survival of ccRCC patients in **(G)** training set, **(H)** testing set and **(I)** whole TCGA-KIRC cohort. PCA showing the ability to distinguish ccRCC patients into high and low-risk categories in **(J)** training set, **(K)** testing set and **(L)** whole TCGA-KIRC cohort.

**Figure 5:**
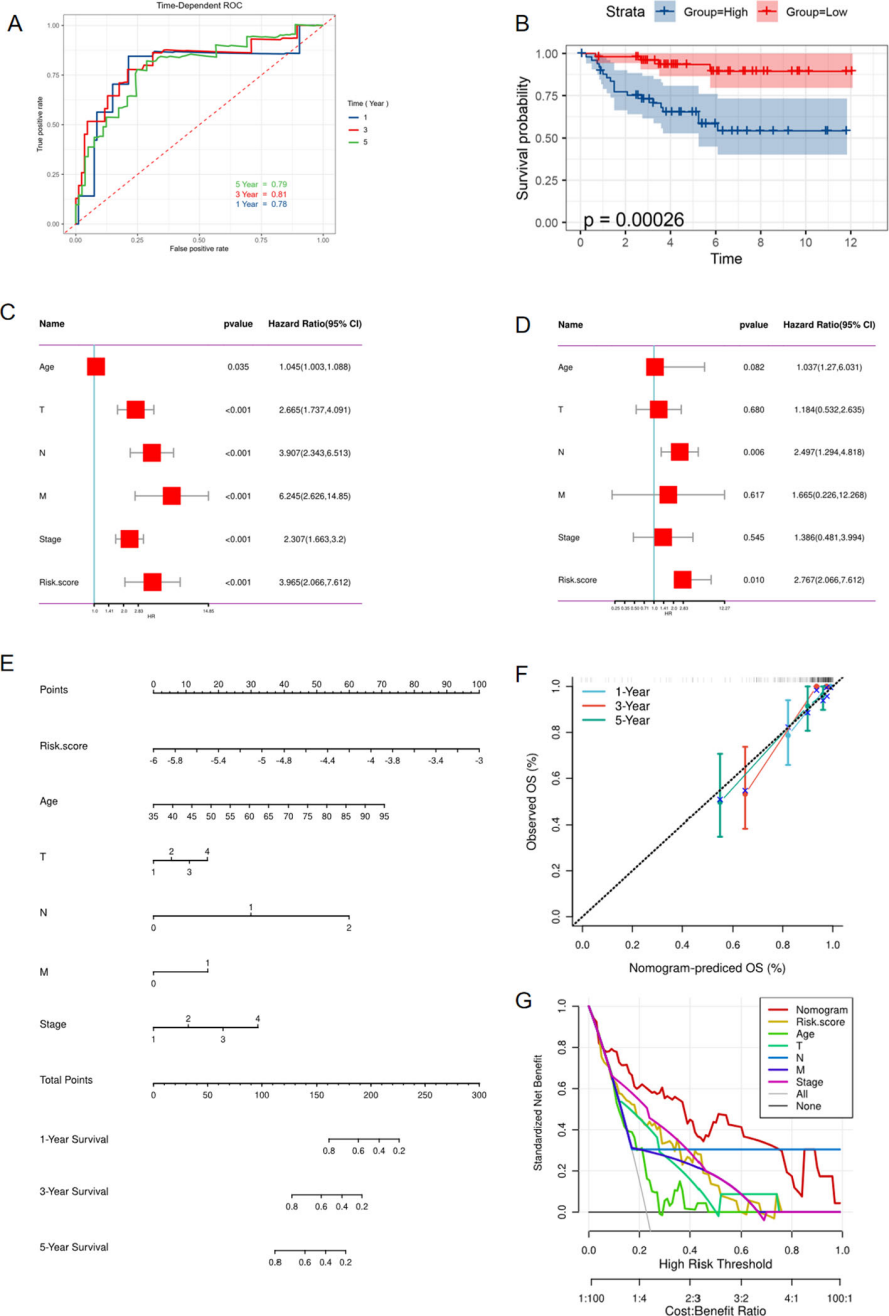
External validation of risk model in E-MTAB-1980 cohort. **(A)** ROC curve showing the predictive value of risk model for 1,3,5 year survival of ccRCC patients. **(B)** Kaplan-Meier curve showing the different overall survival between high and low risk score groups. **(C)** Univariate Cox regression analysis of clinical variables and risk score. **(D)** Multivariate Cox regression showing the independent prognostic value of risk model. **(E)** Nomogram to predict 1-, 3-, and 5-year OS of ccRCC patients. **(F)** Calibration curves to evaluate the accuracy of nomogram in predicting survival rate at 1, 3, and 5 years. **(G)** Decision curve analysis(DCA) curve showing the ability of nomogram in predicting 1-,3-,5- years survival rate of ccRCC patients.

For the sake of constructing a robust prognostic risk model, the TCGA-KIRC cohort was randomly divided into training and testing sets. In the training set, 60 prognostic genes were screened from 68 RAS-related genes (RRGs) through univariate Cox regression analysis (**Supplementary Figure 2**). Afterwards, 60 prognosis genes acquired from last step were recruited for further digging key genes using Lasso regression and Randomforest method respectively, with the former screening out 16 genes (**Figures 3A, B**) and the latter selecting 20 genes (**Figure 3C**) that were more conducive to predicting prognosis of ccRCC. The common genes obtained from both methods were subjected to multivariate Cox regression analysis (**Figure 3D**). Three hub genes SLC6A19, SLC16A12 and SMIM24 were determined, risk score was attained followed the formula: (-0.17×SLC6A19 expression) + (−0.12 × SLC16A12 expression) + (−0.13 × SMIM24 expression). For follow-up analysis, we divided the risk score into high and low groups with the median as the cutoff. Kaplan-Meier curves suggested that the prognosis of the high-risk scoring group was significantly worse than that of the low-risk scoring group in training (**Figure 4A**, P<0.0001), testing (**Figure 4B**, P=0.0014) and whole cohorts (**Figure 4C**, P<0.0001). The risk distribution map showed that higher risk scores correlated with more deaths in ccRCC patients, and the expression of the three hub genes was upregulated in the low-risk group (**Figures 4D-F**). In order to validate the predictive efficiency of the risk model, time-dependent ROC analysis was performed, with areas under the curve (AUC) of 1-year, 3-year, and 5-year being 0.74, 0.73, and 0.74 in the training set (**Figure 4G**), showing a robust prediction ability. Similarly, a high predictive value was observed in the testing set and the entire cohort (**Figures 4H, I**). Principal component analysis (PCA) was applied to confirm that the risk score could significantly distinguish patients, demonstrating that the risk model based on the expression profile of the three hub genes was a strong prognostic marker (**Figures 4J-L**). For further validation of the predictive value of the risk model, external validation was performed in the E-MTAB-1980 cohort. The ROC curve showed AUCs of 1-year, 3-year, and 5-year to be 0.78, 0.81, and 0.79, respectively (**Figure 5A**). Kaplan-Meier curves also manifested that the prognosis of the high-risk group was significantly worse than that of the low-risk group (**Figure 5B**).

### Associations between risk score and clinical characteristics

**Figure 6:**
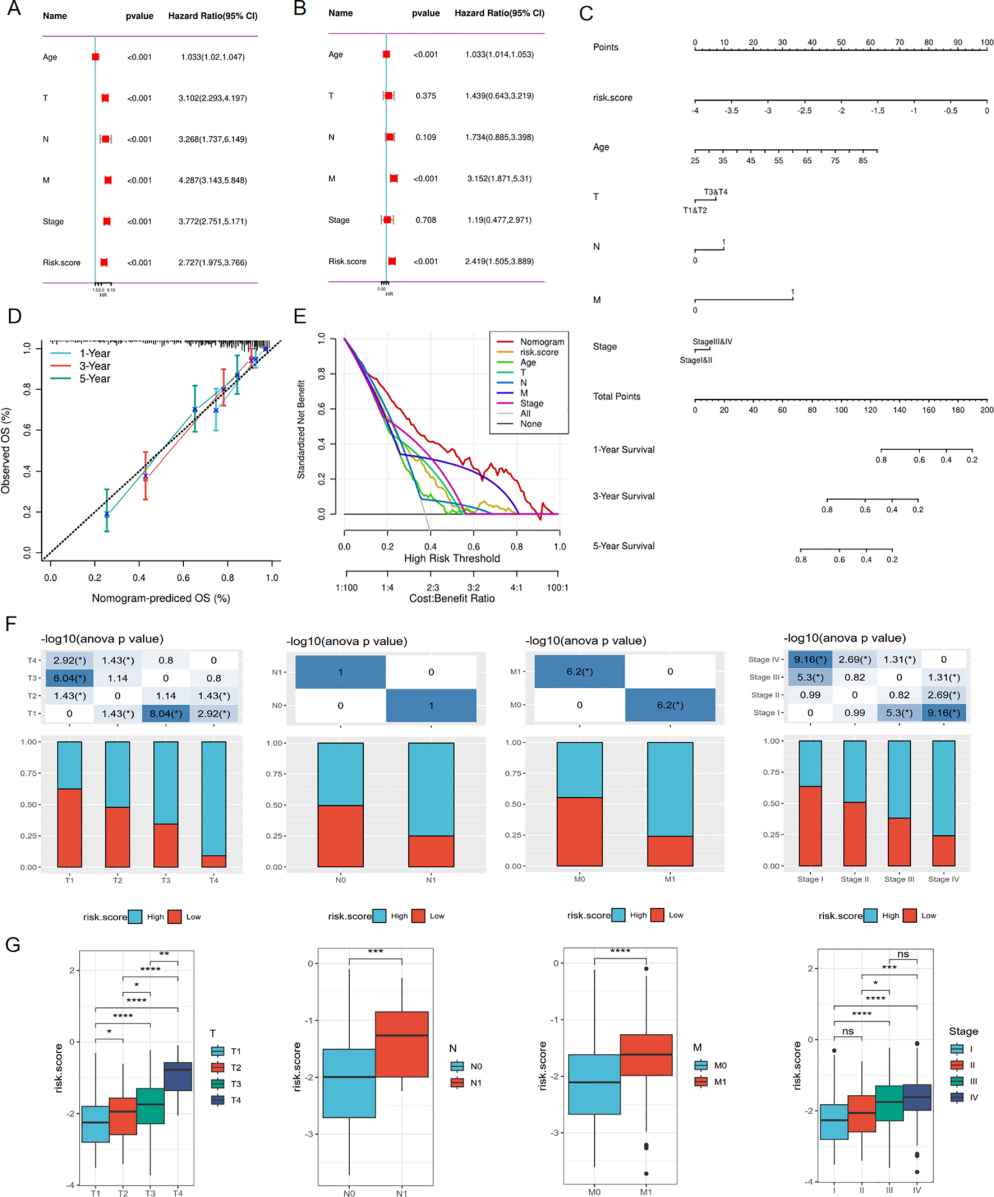
Associations between risk score and clinical characteristics in TCGA cohort. **(A)** Univariate Cox regression analysis of clinical variables and risk score. **(B)** Multivariate Cox regression showing the independent prognostic value of risk model. **(C)** Nomogram to predict 1-, 3-, and 5-year OS of ccRCC patients. **(D)** Calibration curves to evaluate the accuracy of nomogram in predicting survival rate at 1-, 3-, and 5-year. **(E)** Decision curve analysis(DCA) curve showing the ability of nomogram in predicting 1,3,5 years survival rate of ccRCC patients **(F, G)** Correlations between risk score and clinical variables. *p < 0.05, **p < 0.01, ***p < 0.005, ****p < 0.001.

To eliminate the influence of other factors on the risk model and confirm its independent predictive ability, the model and other clinical variables were included in univariate and multivariate Cox regression analyses. The results demonstrated that the risk model could serve as an independent marker to predict the prognosis of ccRCC patients (**Figures 6A, B**). Additionally, we developed a nomogram that estimates the survival probability of patients (**Figure 6C**). Calibration curves and decision curve analysis (DCA) showed the accuracy and predictive ability of the nomogram for 1-, 3-, and 5-year survival rates of ccRCC patients (**Figures 6D, E**). A Chi-square test was used to verify the correlations between the risk score and clinical parameters such as TNM stage, indicating that among patients with a high-risk score, the proportion of patients with advanced clinical stage was higher compared to the low-risk score group (**Figure 6F**). Furthermore, patients were grouped based on stage, and the risk scores between different groups were compared. The findings showed that the risk score was higher in the population with an advanced stage (**Figure 6G**). The univariate and multivariate COX regression also proved that this risk model served as an independent prognostic indicator in the E-MTAB-1980 cohort (**Figures 5C, D**). Moreover, the nomogram model was also constructed in the E-MTAB-1980 cohort (**Figure 5E**), calibration curves and DCA showed the accuracy and ability of this model (**Figures 5F, G**).

### Stratified validation in predictive ability of risk model

**Figure 7:**
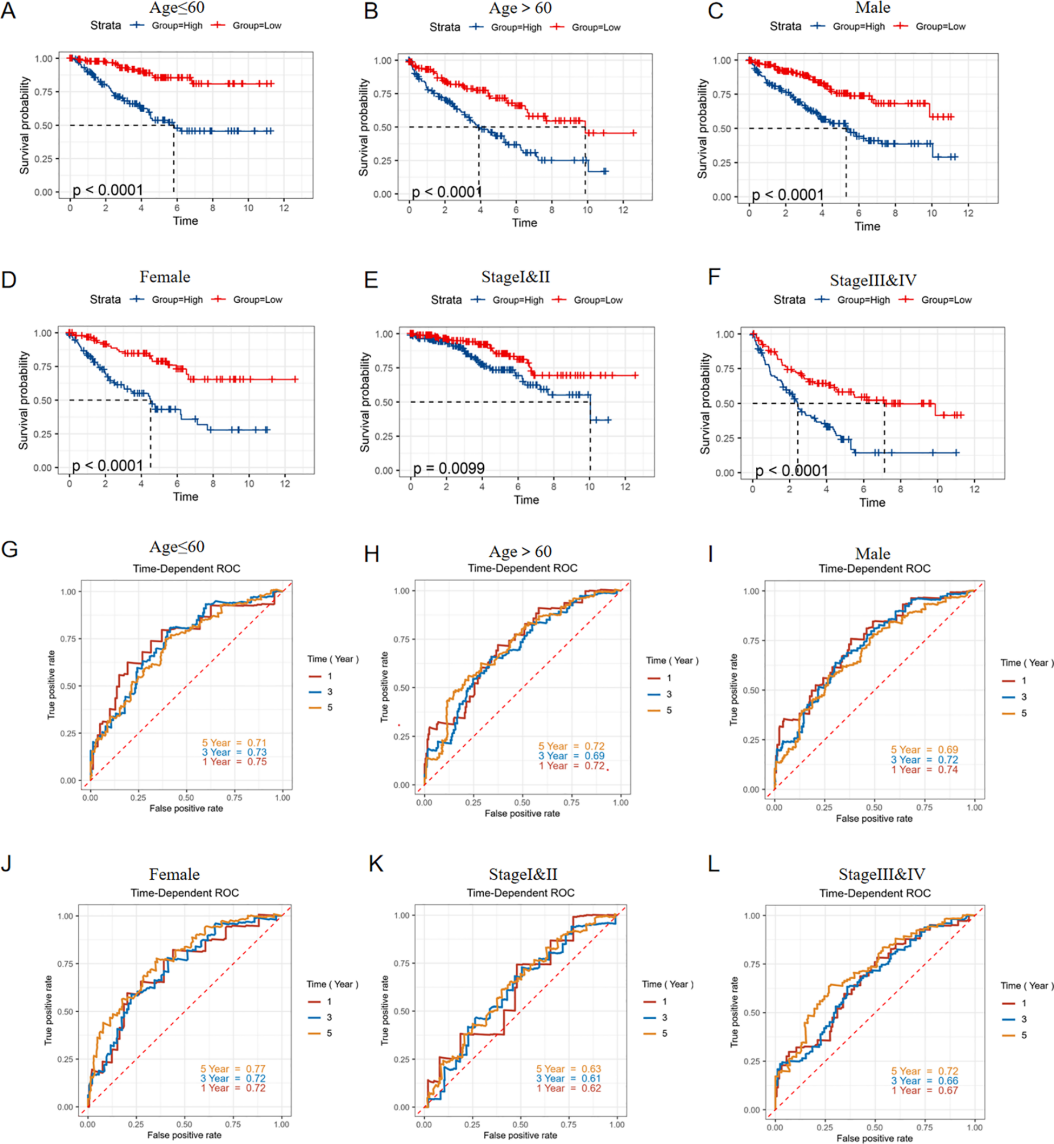
Stratified validation in predictive ability of risk model. KM survival curves predict the OS of the TCGA-KIRC cohort in **(A)** Age ≤ 60years, **(B)** Age > 60years, **(C)** Male, **(D)** Female, **(E)** StageI&II, **(F)** Stage III&IV; ROC curves showed the predictive ability for OS of the TCGA-KIRC cohort in **(G)** Age ≤ 60years, **(H)** Age > 60years, **(I)** Male, **(J)** Female, **(K)** StageI&II, **(L)** Stage III&IV.

To ensure that the predictive power of the risk model was not confounded by variables like gender, age, or clinical stage, we stratified ccRCC subtypes based on age (age ≤60 and age >60) (**Figures 7A, B, G, H**), gender (male and female) (**Figures 7C, D, I, J**), pathological stage(stageI&II and stageIII&IV) (**Figures 7E, F, K, L**). We then performed the Kaplan-Meier and ROC analyses within specific subgroups utilizing OS and results demonstrated that all subgroups showed better OS in the low-risk group and presented appreciable AUC for risk score, which verified the predictive stability of this risk model.

### Mutation landscape and drug prediction of various risk groups

**Figure 8:**
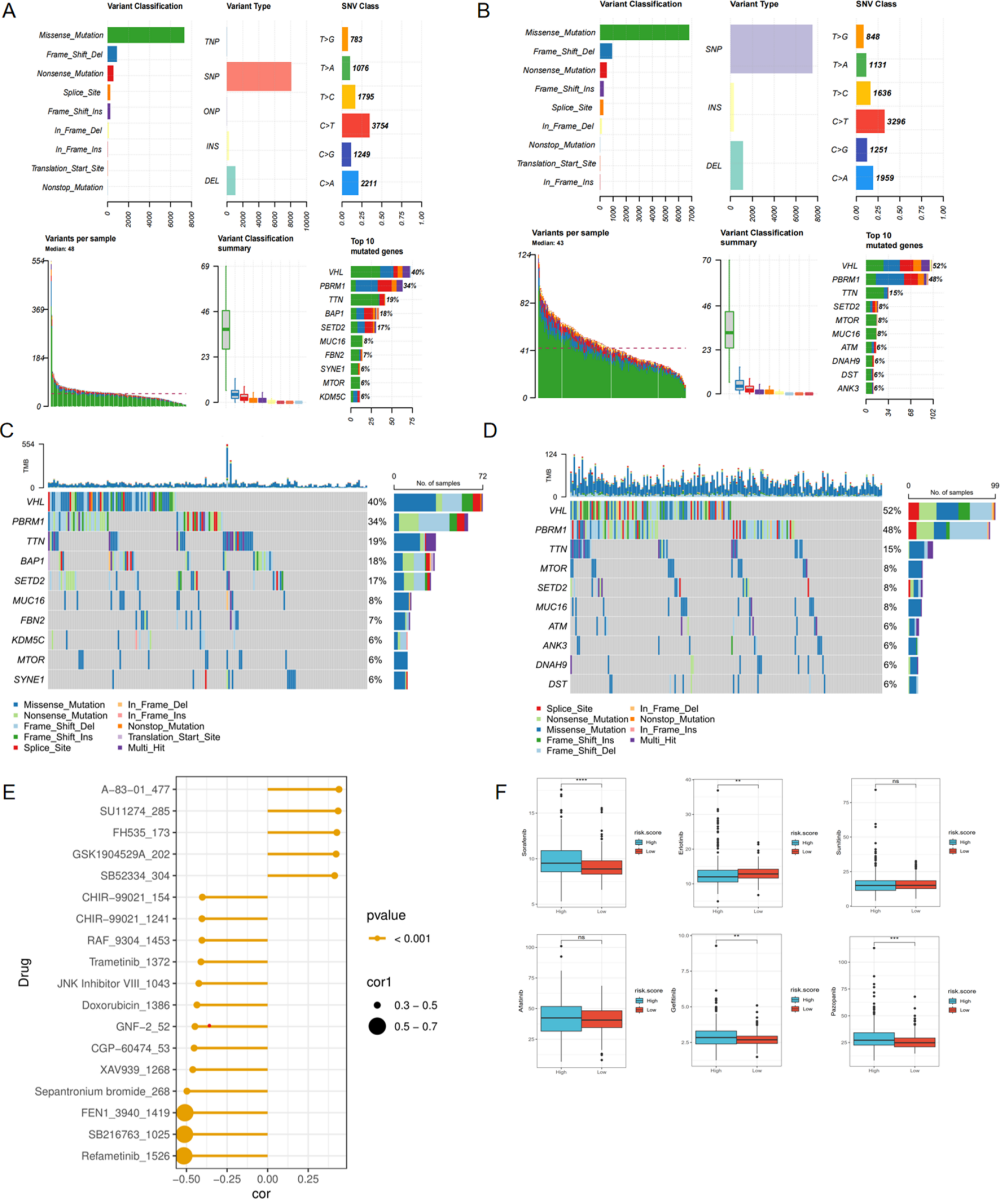
Mutation and drug prediction analyses. **(A)** The summary of the mutation state in high-risk score group. **(B)** The summary of the mutation state in low-risk score group. **(C)** The top 10 most frequently mutated genes in high-risk score group. **(D)** The top 10 most frequently mutated genes in low-risk score group. **(E)** Correlation between drug targets and risk score. **(F)** Comparison of sensitivity of common targeted drugs in different risk groups. *p < 0.05, **p < 0.01, ***p < 0.005, ****p < 0.001. ns, not significant.

The overall mutation condition in high and low-risk groups was analyzed, revealing subtle differences in variant classification, variant type, and SNV class. The top ten most variable genes were extracted and showed differences between each group (**Figures 8A-D**). To predict the response of high-risk patients to various drugs and identify appropriate treatments, drug sensitivity prediction was performed using the ‘oncoPredict’ package, and the IC50 values of different drugs for each patient were ultimately obtained. A correlation analysis between the IC50 value and risk score was primarily conducted, and drugs with absolute correlation coefficients exceeding 0.4 were identified after screening (**Figure 8E**). We then conducted a comparative analysis to assess the varying sensitivities of distinct risk groups to commonly used tyrosine kinase receptor inhibitors (TKIs) in clinical settings. Sorafenib, Gefitinib, and Pazopanib demonstrated higher IC50 values in the high-risk group, indicating less sensitivity to these drugs, while only Erlotinib exhibited a significantly lower IC50 in the high-risk group, showing a stronger sensitivity (**Figure 8F**).

### Enrichment and immune analysis between different risk groups

**Figure 9:**
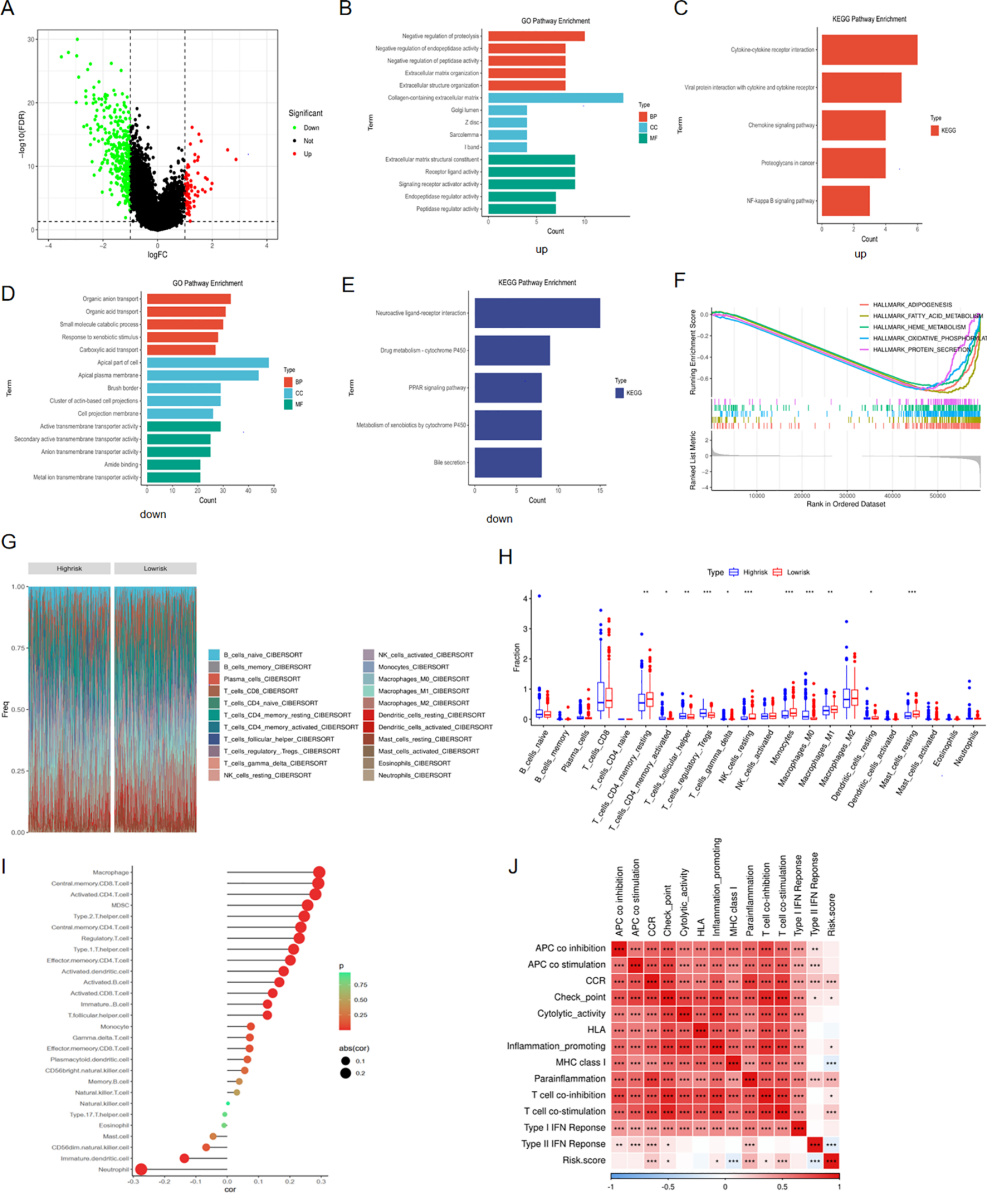
Enrichment and immune analyses. **(A)** DEGs between high and low risk groups; **(B)** GO pathway enrichment of up-regulated DEGs in high-risk group; **(C)** KEGG pathway enrichment of up-regulated DEGs in high-risk group; **(D)** GO pathway enrichment of down-regulated DEGs in high-risk group; **(E)** KEGG pathway enrichment of down-regulated DEGs in high-risk group; **(F)** GSEA analysis for DEGs between high and low risk groups; **(G)** Cibersort analysis showing the proportion of 22 immune cells in various risk groups; **(H)** Differences in infiltration fraction of 22 immune cells among different risk groups; **(I)** Correlation analysis between immune cell infiltration and risk score; **(J)** Heat map showing the correlation between immune pathways and risk score. *p < 0.05, **p < 0.01, ***p < 0.005.

We firstly identified the differentially expressed genes between high and low risk groups (**Figure 9A**). For purpose of digging the underlying signaling pathways influenced by varying degrees of risk, we conducted a series of enrichment analyses. The top five pathways with the highest gene count in ‘Biological Process’, ‘Cellular Component’, and ‘Molecular Function’ categories from the GO analysis, as well as the top five pathways from KEGG, were displayed for both up-regulated and down-regulated DEGs, respectively (**Figures 9B-E**). Furthermore, GSEA analysis was performed using the 50 classic pathways in the HALLMARK model. The results showed that the activity of HALLMARK_ADIPOGENESIS, HALLMARK_FATTY_ACID_METABOLISM, HALLMARK_HEME_METABOLISM, HALLMARK_OXIDATIVE_PHOSPHORYLATION, and HALLMARK_GLYCOLYSIS was significantly reduced in the high-risk group (**Figure 9F**). The infiltration fraction of 22 immune cells for each patient in the TCGA-KIRC cohort was calculated using the CIBERSORT method. A number of these cells showed differences in infiltration degree between high and low-risk groups. Among them, T regulatory cells (Tregs), resting natural killer (NK) cells, monocytes, and M0 macrophages exhibited the most significant differences (**Figures 9G, H**). Furthermore, we characterized 28 immune cell types and 13 immune functions from the literature. Using ssGSEA, we quantified their respective scores in each patient based on the expression profiles of characteristic genes. Subsequently, we performed a Spearman correlation analysis to examine the relationship between these immune scores and the patients’ risk scores. We observed a positive correlation between the infiltration levels of the majority of immune cells and the associated risk (**Figure 9I**). The correlation heatmap indicated that immune pathways such as CCR, Checkpoint, Inflammation-promoting, Parainflammation, T-cell co-stimulation, and T-cell co-inhibition were positively correlated with risk scores. In contrast, MHC Class I and Type II IFN response showed an opposite trend (**Figure 9J**).

### Singel-cell analysis for ccRCC and normal samples

**Figure 10:**
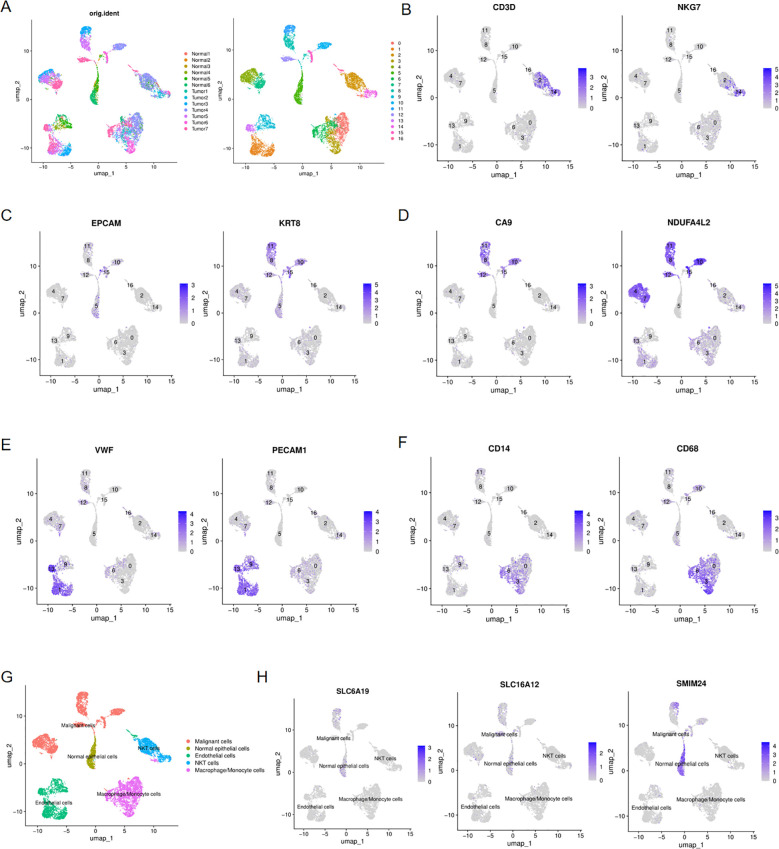
Single-cell analysis of ccRCC and normal kidney tissues. **(A)** UMAP plots showing 17 cell clustering, colored with 7 ccRCC and 6 normal kidney samples and with 17 cell clusters; **(B)** Expression of NKT cells markers in 17 cell clusters; **(C)** Expression of epithelial cell markers in 17 cell clusters; **(D)** Expression of ccRCC cell markers in 17 cell clusters; **(E)** Expression of endothelial cells markers in 17 cell clusters; **(F)** Expression of macrophage/monocyte cells markers in 17 cell clusters; **(G)** UMAP plots showing the five distinct cell types in ccRCC and normal kidney samples;**(H)** UMAP plots showing the expression of SLC6A19, SLC16A12 and SMIM24 in five distinct cell types.

For purpose of investigating the expression of three pub genes in tumor and normal tissue, we performed single-cell analysis to identify their distribution within cell level. By integrating 7 ccRCC samples and 6 normal samples from the GSE159115 dataset, 17 cell clusters were ultimately identified (**Figure 10A**). We subsequently annotated the cell clusters based on the expression of classical cell marker genes. CD30, NKG7 were major cell markers of NKT cell and they were mainly distributed in clusters 2 and 14(**Figure 10B**). EPCAM and KRT8 were signatures of epithelial cell and they presented in clusters 5, 8, 10, 11, 12 and 15(**Figure 10C**). CA9 and NDUFA4L2 served as marker genes of ccRCC cells and they were distributed in clusters 4, 7, 8, 10, 11, 12, 15, which overlapped with the distribution of epithelial cell markers and this resulted from that ccRCC originates from renal tubular epithelial cells (**Figure 10D**). Therefore, only cluster5 was not within the overlap and we preliminarily labeled 5 as normal epithelial cells and defined other cell populations as malignant cells. Furthermore, we identified other two cell populations endothelial cells and macrophage/monocyte cells utilizing their typical markers VWF/PECAM1 and CD16/CD68 respectively (**Figures 10E, F**). Overall, five distinct cell types were eventually categorized (Fig10G). Then, we examined the distribution of SLC6A19, SLC16A12 and SMIM24 in five cell populations and found that they were scattered in normal epithelial and malignant cells, where the former showed more obvious expression (**Figure 10H**).

### The role of SLC6A19 in ccRCC

**Figure 11:**
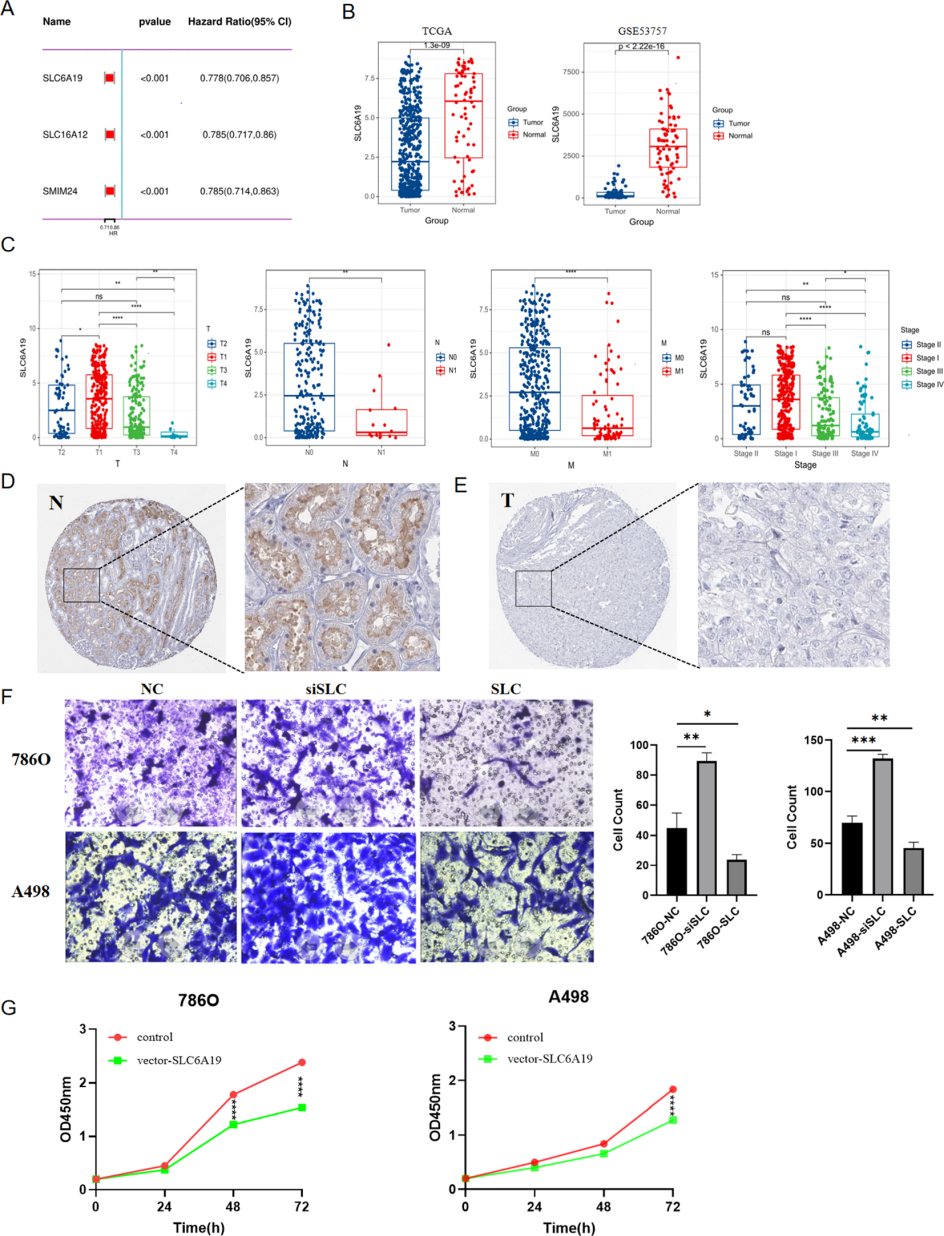
The role of SLC6A19 in ccRCC; **(A)** Univariate Cox regression for SLC6A19, SLC6A12 and SMIM24. **(B)** Differential expression of SLC6A19 between tumor and normal tissues in TCGA,GSE53757; **(C)** Differential expression of SLC6A19 in various clinical stages; **(D)** Immunohistochemical result from the HPA database showing SLC6A19 expression in normal tissue; **(E)** Immunohistochemical result from the HPA database showing SLC6A19 expression in ccRCC tissue; **(F)** Transwell assay showing the impact of SLC6A19 to invasive ability of 786O and A498 cell lines; **(G)** CCK8 assay showing the impact of SLC6A19 to proliferation of 786O and A498 cell lines. *p < 0.05, **p < 0.01, ***p < 0.005, ****p < 0.001. ns, not significant.

Univariate Cox regression analysis indicated that among the three hub genes, SLC6A19 had the most significant impact on the prognosis of ccRCC patients (**Figure 11A**), Therefore, we conducted a series of analyses to investigate the function of SLC6A19 in ccRCC. First, we conducted a comparative analysis of SLC6A19 gene expression levels between tumor and normal tissues across two distinct databases: TCGA and GSE53757. The findings revealed a significant downregulation of SLC6A19 in tumor tissues (**Figure 11B**). Next, we explored the association between SLC6A19 expression and the clinical staging of ccRCC and concluded that the gene was significantly downregulated in the advanced stages of the disease (**Figure 11C**). To verify the expression difference of SLC6A19 between normal and ccRCC tissues, we downloaded immunohistochemical data from the HPA database, and the results showed that SLC6A19 expression was significantly higher in normal tissue and reduced in ccRCC tissue (**Figures 11D, E**). Through transwell assays, we found that the 786O and A498 cell lines with downregulated SLC6A19 exhibited stronger invasiveness, while those overexpressing SLC6A19 showed weaker penetration ability (**Figure 11F**). Moreover, the results of CCK8 assay indicated that SLC6A19 may suppress the proliferation of ccRCC(**Figure 11G**). Additionally, we also elucidated the correlation between SLC16A12&SMIM24 and clinical stage of ccRCC, results showed that in the patients with advanced stage including distant metastasis or lymph node metastasis, both two genes demonstrated lower expression (**Supplementary Figure 5**).

### Identification of downstream pathways of SLC6A19 via GSEA analysis

**Figure 12:**
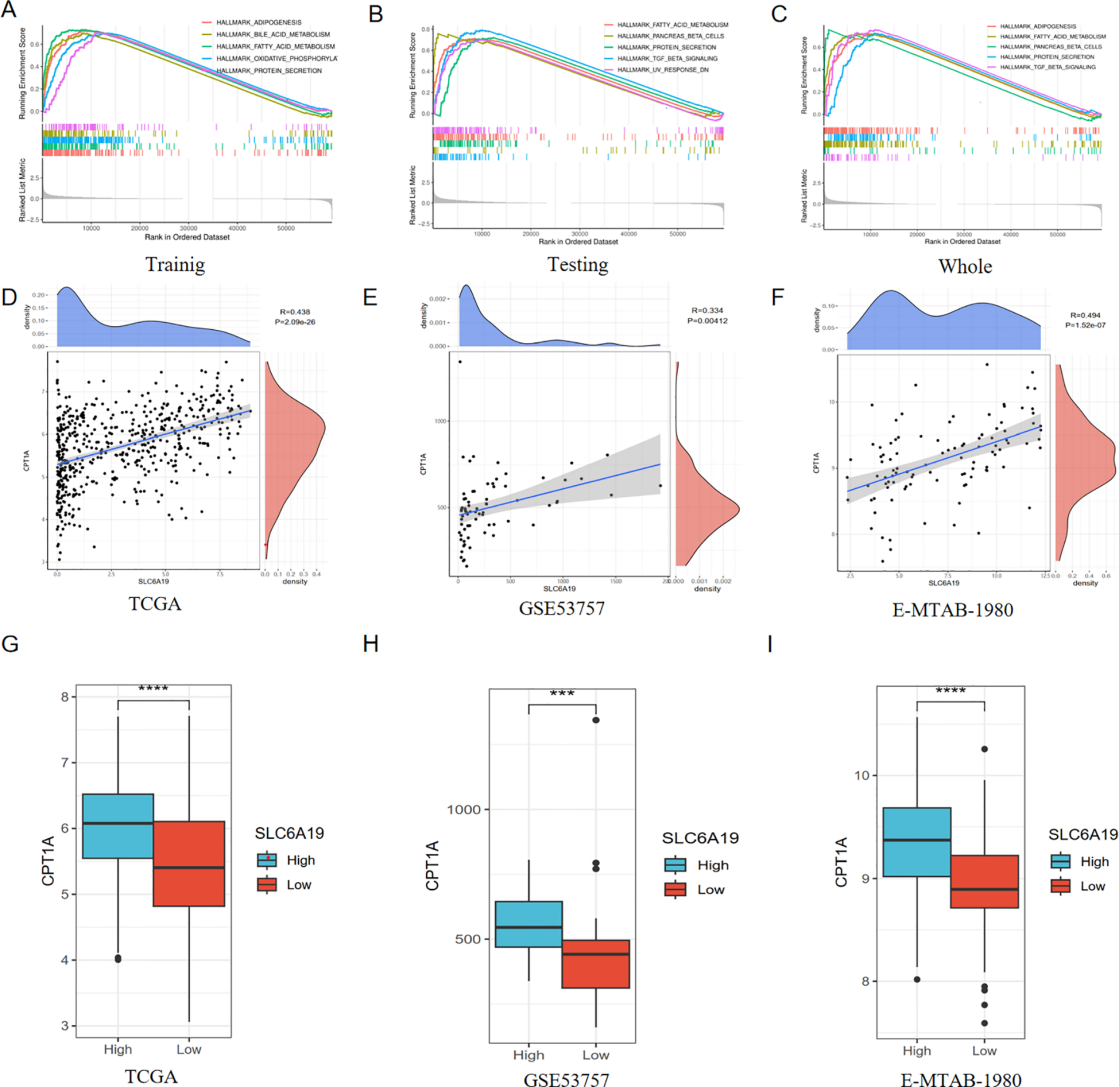
Correlation between SLC6A19 and fatty acid metabolism pathway. GESA presenting SLC6A19 involved pathways in training **(A)**, testing **(B)** and whole TCGA **(C)** sets; Correlation analysis between SLC6A19 and CPT1A in TCGA **(D)**, GSE53757 **(E)** and E-MTAB-1980 **(F)** cohorts; Differential expression of CPT1A between high and low SLC6A19 groups in TCGA **(G)**, GSE53757 **(H)** and E-MTAB-1980 **(I)** cohorts. ***p < 0.005, ****p < 0.001.

To further explore the underlying mechanisms of SLC6A19 in modulating ccRCC, we employed the GSEA method to identify pathways closely correlated with SLC6A19. Through cross-validation with training, testing, and the entire TCGA dataset, we found that the fatty acid metabolism pathway was significantly enriched (**Figures 12A-C**). For further verifying this finding, we performed the GSEA analysis utilizing three external datasets, and the results consistently revealed that SLC6A19 was closely associated with fatty acid metabolism (**Supplementary Figure 3**). We next investigated the relationship between SLC6A19 and CPT1A, a key enzyme in fatty acid beta-oxidation, the inactivation of which could contribute to the progression of ccRCC. Spearman correlation analysis demonstrated that CPT1A expression was strongly positively correlated with SLC6A19 in the TCGA dataset, a finding that was further validated in the GSE53757 and E-MTAB-1980 cohorts (**Figures 12D-F**). Subsequently, we divided samples from the aforementioned cohorts into high and low SLC6A19 expression groups and compared the expression levels of CPT1A between these groups. Box plots indicated that when SLC6A19 expression was high, CPT1A expression also increased, suggesting that the two genes may have a synergistic function (**Figures 12G-I**).

## Discussion

It is a widely accepted belief was that the tumorgenesis of ccRCC resulted from disorder of multiple pathway mainly including the dysregulation of von hippel-lindau/HIF protein signaling and PI3K/AKT/mTOR, the deletion of 3P chromosome and the defection of homologous recombination repair (21–24). Moreover, the progress of ccRCC was also intimately associated with various aberrant metabolisms such us the reprogramming of lactate metabolism, glycolysis, tricarboxylic acid cycle, pentose phosphate pathway, fatty acid synthesis and degradation, tryptopha and arginine metabolism (25). Recently, more and more evidences indicated that the imbalance of RAS regulating blood volume and blood pressure was intimately linked to the etiology and progression of malignancies, especially was extensively studied in breast and lung cancer (8, 12, 25–28). Recent studies have documented a correlation between RAS and urogenital malignancies. Specifically, they have observed that under the long-term influence of AngII, normal prostate cells can exhibit hypertrophy and hyperplasia and extracellular matrix (ECM) was more susceptible to degradation by the upregulation of MMP-2 and MMP-9, suggesting a potential role of RAS in the pathogenesis of prostate cancer (29). In bladder cancer, the expression of the angiotensin II type 2 receptor (AT2R) is downregulated and it has been demonstrated that AT2R can suppress tumor growth and angiogenesis by inactivating the extracellular signal-regulated kinase (ERK) pathway, which in turn leads to a reduction in vascular endothelial growth factor (VEGF) production (30).Indeed, several studies have detailed the role of intrarenal renin-angiotensin system (iRAS) in regulating angiogenesis, cell differentiation, and proliferation. Moreover, the dysregulation of four pivotal enzymes within iRAS, neutral endopeptidase (NEP/CD10), angiotensin-converting enzyme-2 (ACE2), angiotensin-converting enzyme(ACE) and aminopeptidase A (APA) were considered to be intimately linked to the progression of renal malignancies (31). Herein, we screened 17 characteristic genes of RAS and preliminarily elucidated their correlation with ccRCC, subsequently, we constructed a robust survival prognosis model employing a suite of bioinformatics methods majorly including WGCNA, ssGSEA, Lasso and Randomforest analyses, offering enhanced predictive accuracy for patient outcomes.

RAS encompasses multiple axes, among which the AngII signaling through its receptors AGTR1 and AGTR2 has been the subject of extensive research, where this axis is known to intersect with a variety of pathways implicated in tumorigenesis, highlighting its potential role in cancer biology. AGTR1 and AGTR2 are fundamentally G protein-coupled receptors (GPCRs). Upon binding of AngII to AGTR1, it induces conformational alterations in the associated G protein, which triggers a cascade of intracellular signaling events, initiating a multitude of downstream pathways that regulate diverse physiological responses, including activation of MAPK, c-Src, Tyk2, Pyk2, Jak2, p21Ras, Akt and receptor tyrosine kinases (32). Currently, AGTR1 has been identified as a protumorigenic factor, with its oncogenic properties primarily manifested in the following aspects: (i) facilitating cell proliferation and angiogenesis by activating PI3K/AKT signaling cascade and increasing both VEGF and VEGF receptor 2 expression in endothelial cells (33); (ii)inhibiting cell apoptosis in a manner of NF-κB activation (26); (iii) inducing cell migration, invasion, and metastasis by increasing expression of endothelial adhesion molecules like E-selectin, P-selectin and vascular cell adhesion molecule-1 (VCAM-1) (34). Conversely, AGTR2 can exert anti-tumor effects predominantly through its antagonistic action on angiogenesis (35). The ACE2-Ang-(1–7)-MasR axis, which represents a newly discovered component of the RAS, has been shown to be up-regulated or down-regulated in different cancers (36). It had been suggested that ACE2 expression is decreased in breast cancer, NSCLC, hepatocellular carcinoma and pancreatic cancer (37–40). The MasR has been found to be significantly up-regulated in colon cancer tissues and in association with colorectal cancer metastasis compared with levels in non-neoplastic colon mucosal tissue (41).

Our study culminated in the identification of three key RAS-related genes—specifically SLC6A19, SLC16A12, and SMIM24, which have been demonstrated to be significantly correlated with the prognosis of ccRCC and utilizing these genes, we developed a robust prognostic model. Among these trio of genes, SLC16A12 has garnered considerable attention in the context of ccRCC, with a substantial body of research dedicated to understanding its role (42–44). In contrast, the remaining two genes, SLC6A19 and SMIM24, have received significantly less attention in tumor-related research, with scant reports on their potential implications in oncology. SLC6A19, functioning as a sodium-coupled neutral amino acid transporter, plays a pivotal role in the intestinal absorption of amino acids derived from dietary proteins and in the renal reabsorption of filtered amino acids (45).In one article, a decrease in SLC6A19 expression within ccRCC tumor tissues was noted, corroborating the findings of our research. While it is imperative to emphasize that our research has dug the underlying pathway about SLC6A19 and identified a robust correlation between this gene and fatty acid metabolism in ccRCC, which was initially uncovered through GSEA and further substantiated by subsequent experimental validations. With regard to SMIM24,it was considered as a kind of membrane component and mainly mentioned in Alzheimer disease (46).

In the concluding segment of the article, the pivotal role of SLC6A19 in clear cell renal cell carcinoma (ccRCC) is underscored, highlighting its intriguing interplay with fatty acid metabolism. This discovery not only deepens our understanding of ccRCC’s pathophysiology but also presents potential avenues for therapeutic intervention. Generally, ccRCC is histologically distinguished by the presence of lipid droplets within the cytoplasm, with fatty acids being the predominant constituents of these deposits and the abnormalities in certain pivotal enzymes within the fatty acid metabolism pathway may exert profound influence on the development of ccRCC (47). Carnitine palmitoyltransferase 1A (CPT1A) is a one of the most crucial components in fatty acid metabolism, serving as the rate-limiting enzyme in the β-oxidation pathway, its deficiency can result in a range of metabolic disorders, including hypoketotic hypoglycemia, hepatomegaly, increased concentrations of circulating free fatty acid and the abnormal deposition of lipids within skeletal muscle tissue (48). Research has established that the expression of CPT1A is significantly downregulated in ccRCC tumor tissues and patients exhibiting reduced CPT1A levels were frequently correlated with a poorer OS (49, 50). Emerging data indicated that the enhancing CPT1A activity could potentially represent a novel therapeutic target for the management of ccRCC (41).

It is noteworthy to highlight that our research has uncovered a significant positive correlation between the expression levels of SLC6A19 and CPT1A, a finding that has been consistently validated across multiple cohorts. Western blot provided compelling evidence that the downregulation of SLC6A19 results in a concomitant reduction in CPT1A expression. This discovery not only strengthens the link between these two proteins but also suggests a regulatory interplay that could have profound implications for the understanding of ccRCC’s metabolic pathways and the development of targeted therapies.

## Conclusion

Firstly, the risk model that was established based on RAS related genes can availably predict the prognosis of ccRCC patients and accurately forecast the responsiveness of patients to various commonly utilized targeted therapies in clinical settings. Secondly, SLC6A19 plays a significant role in ccRCC and presents intimately correlation with fatty acid metabolism alongside with CPT1A, which is extensively considered as a tumor suppressor of ccRCC.

## Data Availability

The original contributions presented in the study are included in the article/**Supplementary Material**. Further inquiries can be directed to the corresponding authors.

## Supplementary material

Supplementary Figure 1 Kaplan-Meier curve showing the impact of 17 RAS signature genes on survival of ccRCC patients.

Supplementary Figure 2 Univariate Cox regression for 68 RAS related genes.

Supplementary Figure 3 GSEA analysis for investigating correlation between SLC6A19 and fatty acid metabolism pathway.

Supplementary Figure 4 Quality control of sc-RNA analysis.

Supplementary Figure 5 Correlation between SLC16A12&SMIM24 and clinical stage of ccRCC.
