# Supplementary material for: Identification of a novel prognostic and therapeutic prediction model in clear cell renal carcinoma based on Renin-angiotensin system related genes
Source: Front Endocrinol (Lausanne). 2025 Mar 3;16:1521940. doi: 10.3389/fendo.2025.1521940 (PMC11911175; doi:10.3389/fendo.2025.1521940)
Supplement: Supplementary Figure 1 — Kaplan-Meier curve showing the impact of 17 RAS signature genes on survival of ccRCC patients. [file DataSheet1.pdf]

Clinical data in TCGA-KIRC

Characteristics	High risk	Low risk
**Age**		
≤60	125	139
＞60	141	127
**Gender**		
Male	191	154
Female	75	112
**T stage**		
T1	102	170
T2	36	33
T3	118	62
T4	10	1
**N stage**		
N0	121	119
N1	12	4
**M stage**		
Mo	188	233
M1	60	19
**Clinical stage**		
I	97	169
II	28	29
III	76	47
IV	63	20

Clinical data in E-MTAB-1980

**Characteristics**	High risk	Low risk
**Age**		
≤60	21	23
＞60	29	28
**Gender**		
Male	44	33
Female	6	18
**T stage**		
T1	31	37
T2	5	6
T3	13	7
T4	1	1
**N stage**		
N0	44	50
N1	2	1
N2	4	0
**M stage**		
Mo	42	47
M1	8	4
**Clinical stage**		
I	29	37
II	4	6
III	9	4
IV	8	4
