# Supplementary figures and images for: Identification of a novel prognostic and therapeutic prediction model in clear cell renal carcinoma based on Renin-angiotensin system related genes
Source: Front Endocrinol (Lausanne). 2025 Mar 3;16:1521940. doi: 10.3389/fendo.2025.1521940 (PMC11911175; doi:10.3389/fendo.2025.1521940)

# Supplementary figure1

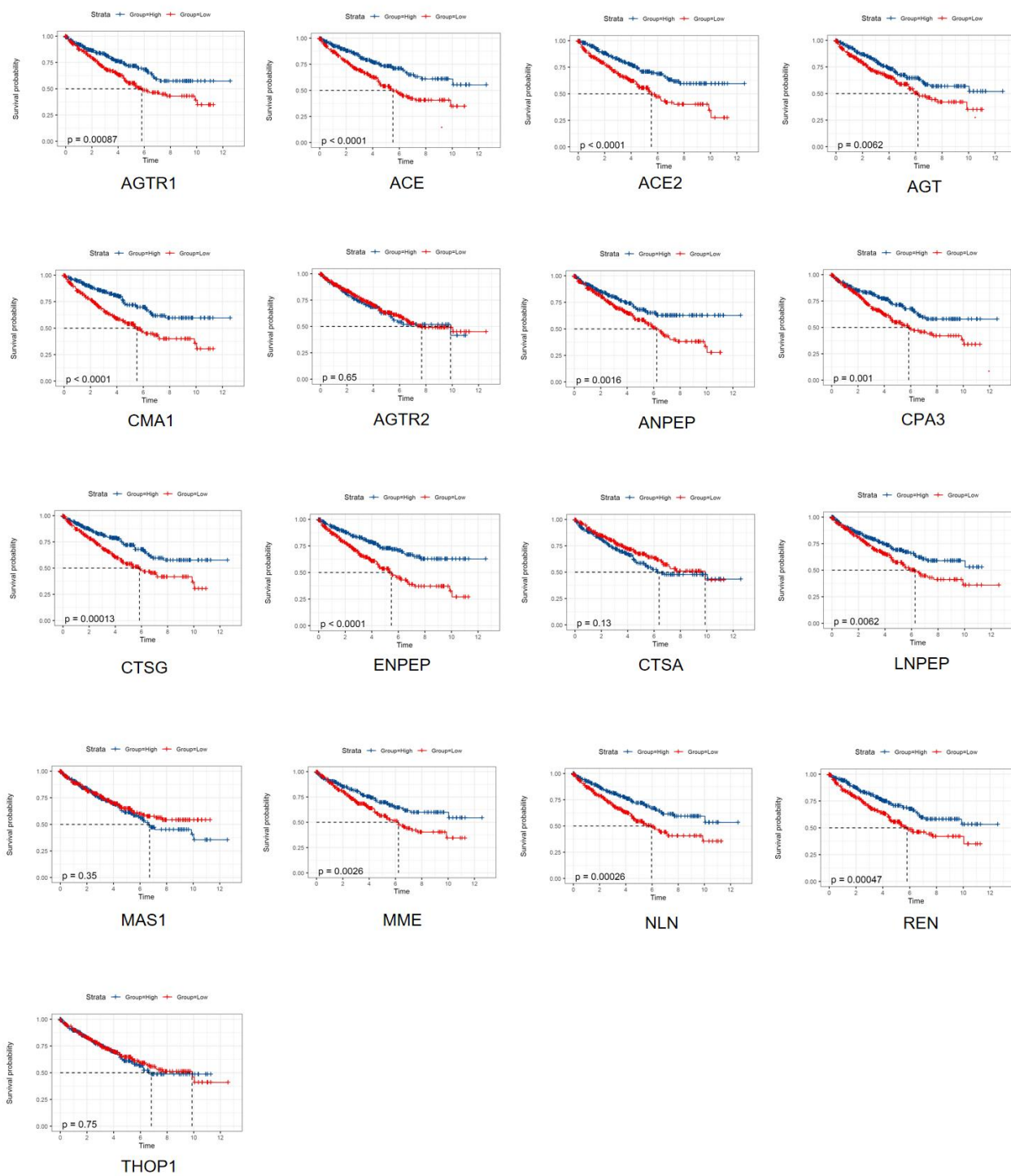

## Supplementary figure2

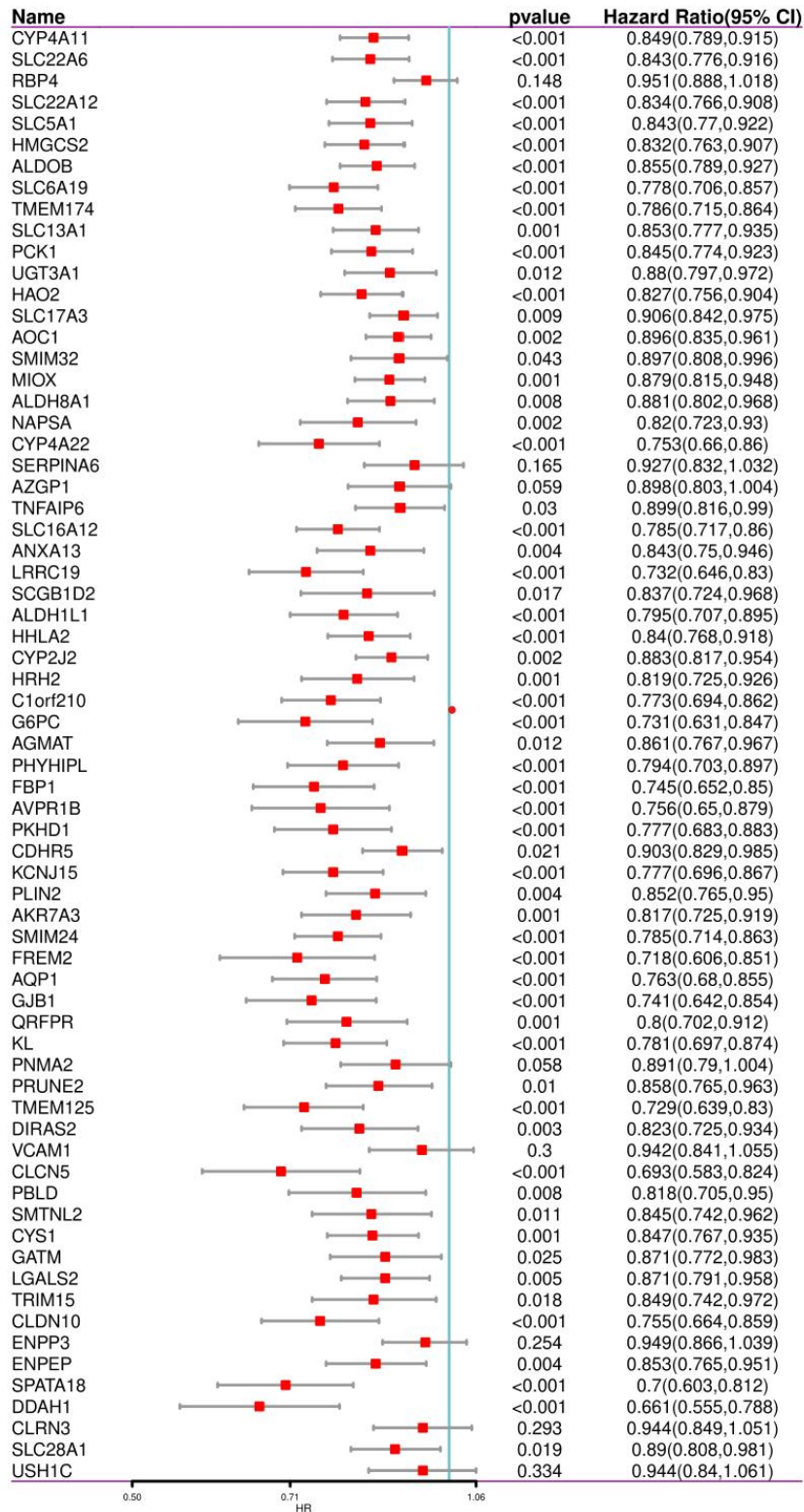

Supplement: Supplementary Figure 2 — Univariate Cox regression for 68 RAS related genes. [file DataSheet2.pdf]
