# Supplementary material for: Identification of a novel prognostic and therapeutic prediction model in clear cell renal carcinoma based on Renin-angiotensin system related genes
Source: Front Endocrinol (Lausanne). 2025 Mar 3;16:1521940. doi: 10.3389/fendo.2025.1521940 (PMC11911175; doi:10.3389/fendo.2025.1521940)

Supplementary figure3

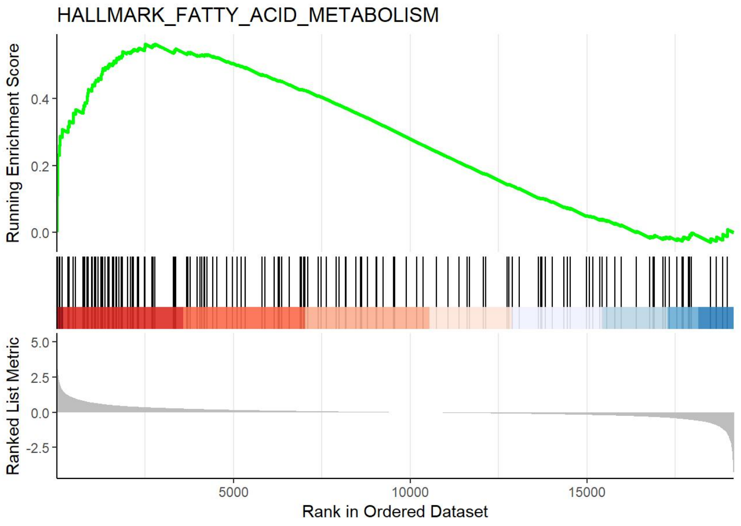

E-MTAB-1980

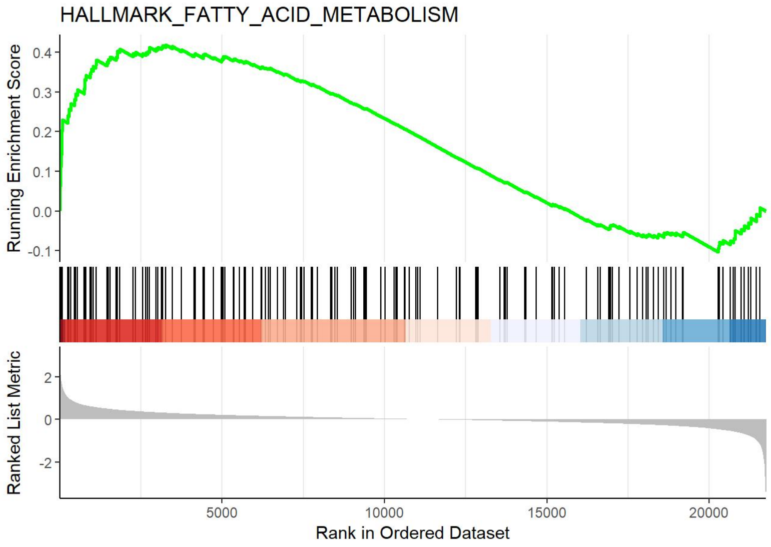

GSE53757

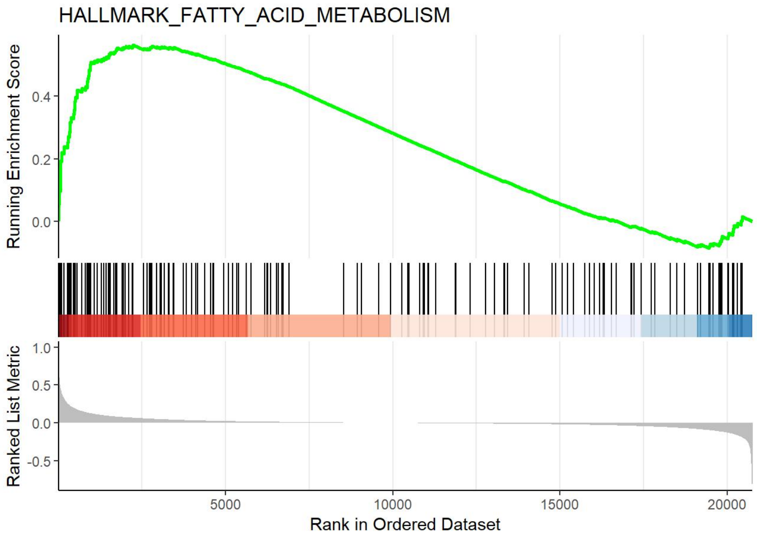

GSE40435

Supplementary figure4

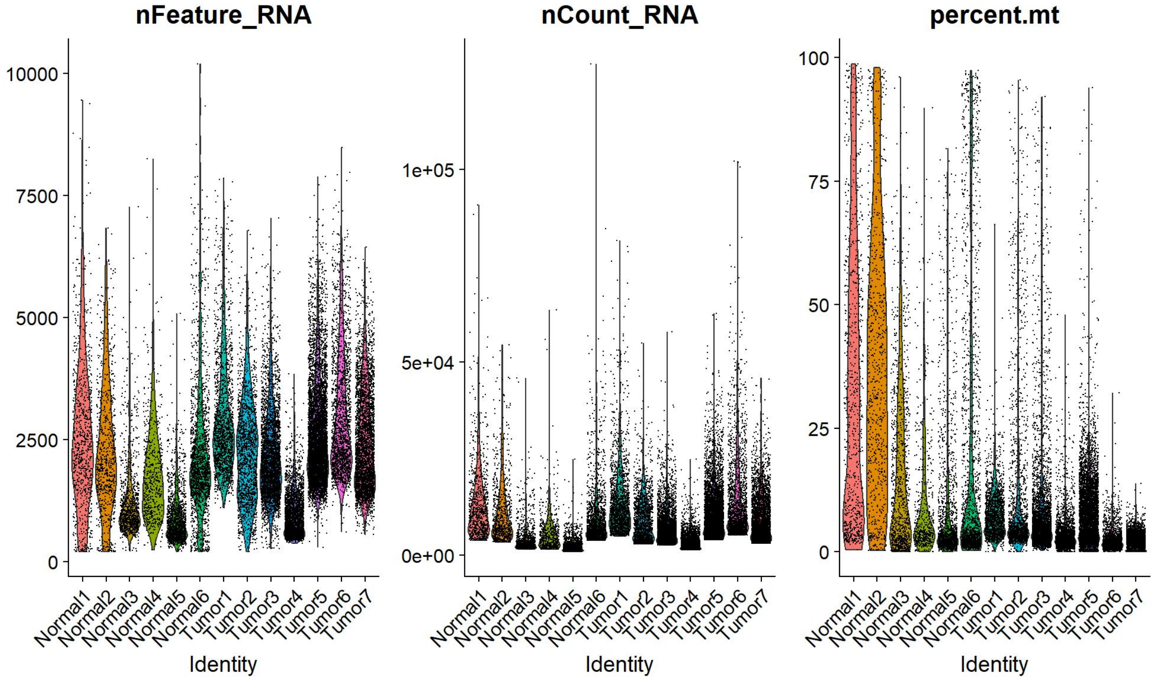

Before quality control

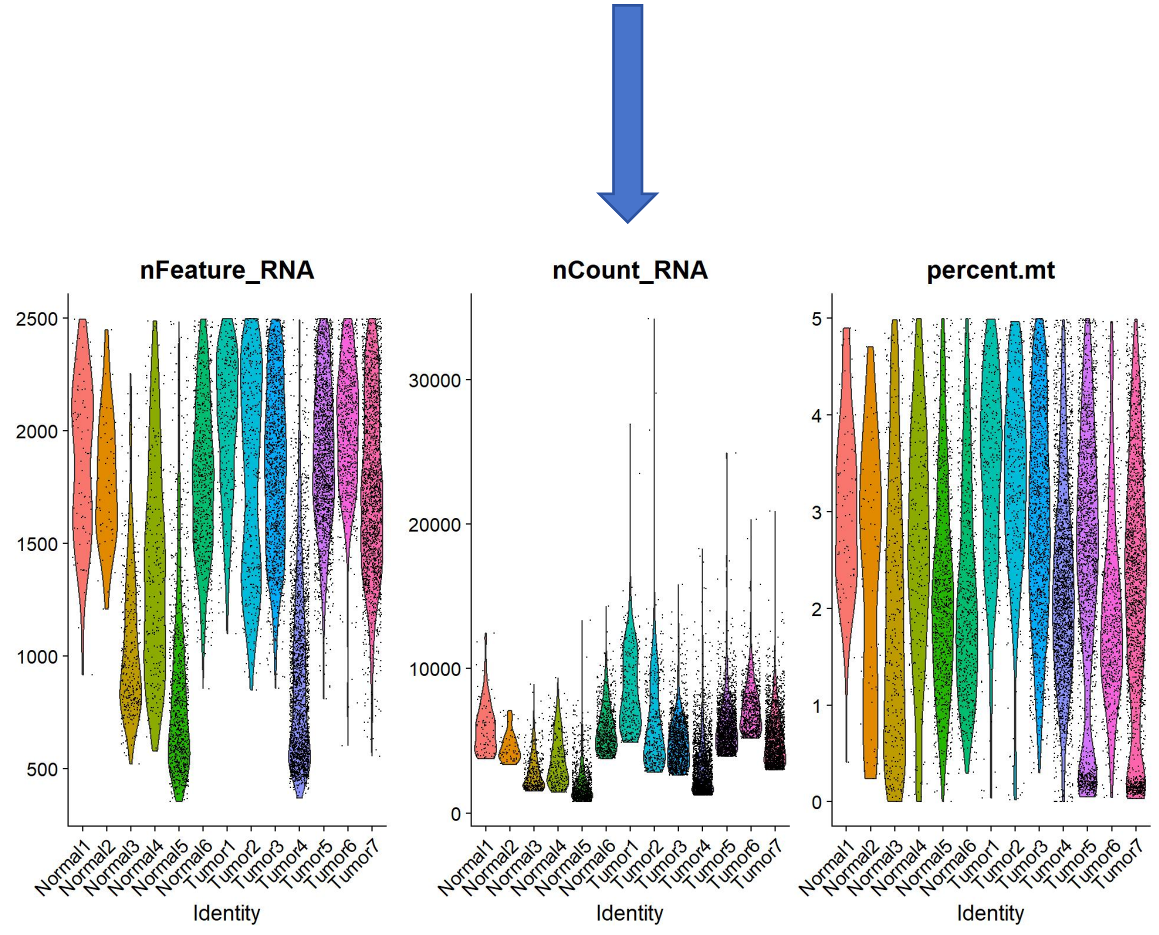

After quality control

Supplementary figure5

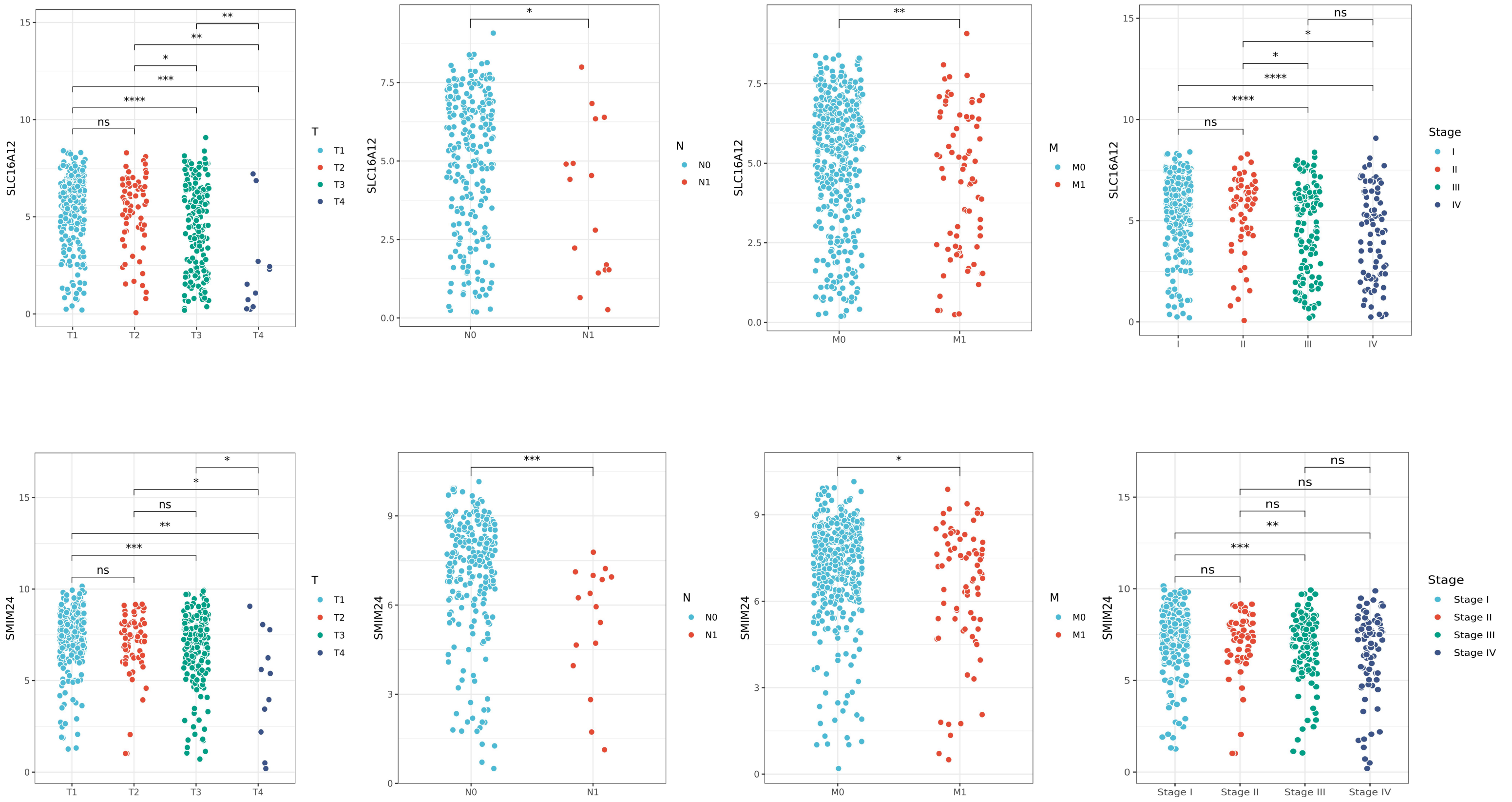

Supplement: Supplementary Figure 3 — GSEA analysis for investigating correlation between SLC6A19 and fatty acid metabolism pathway. [file DataSheet3.pdf]
